# Thermally Induced Reassembly of Ginger Extracellular Vesicles for Oral Therapy of Intestinal Inflammation

**DOI:** 10.34133/research.1377

**Published:** 2026-08-03

**Authors:** Linhai Hou, Jie Cao, Shengjie Gao, Xiaofan Wang, Zhongxian Zhang, Meiqi Li, Yu Mao, Changhong Liu, Ling Yan, Haiping Hao, Lei Zheng

**Affiliations:** ^1^ School of Food and Biological Engineering, Hefei University of Technology, Hefei 230009, China.; ^2^ Engineering Research Center of Bio-Process, Ministry of Education, School of Food and Biological Engineering, Hefei University of Technology, Hefei 230009, China.; ^3^ State Key Laboratory of Natural Medicines, Key Laboratory of Drug Metabolism, China Pharmaceutical University, Nanjing, China.

## Abstract

Plant-derived extracellular vesicles are promising candidates for oral drug delivery, yet their clinical translation is hindered by limited targeting precision and inconsistent systemic absorption. While surface engineering can enhance tissue accumulation, strategies that preserve biocompatibility and enable scalable production remain limited. Here, we introduce boiling as a simple thermal processing approach that structurally reconfigures ginger extracellular vesicles (GEVs) into functionally enhanced, thermally reassembled GEVs (T-GEVs). The surface architecture of T-GEVs is enriched with key vesicle trafficking regulators, including V-type proton adenosine triphosphatase subunit G, ARF1, and β-adaptin-like protein. This specific composition drives their tissue-specific accumulation in the intestine and liver and potentiates clathrin-dependent cellular uptake in intestinal cells by 8.57-fold. Beyond superior intrinsic anti-inflammatory activity through NLRP3 inflammasome suppression, T-GEVs function as an efficient oral delivery platform. When loaded with tumor necrosis factor-α (TNF-α) small interfering RNA, they enable a synergistic therapy that combines innate anti-inflammatory activity with targeted gene silencing of TNF-α, showing potent efficacy in colitis. Our findings position boiling as a natural strategy for enhancing the bioactivity and targeted oral delivery potential of GEVs.

## Introduction

Extracellular vesicles (EVs) are key mediators of intercellular communication and have gained increasing interest as potential therapeutic vehicles [1]. Nature provides a rich source of inspiration for optimizing their delivery, including EVs derived from edible plants, which have evolved to facilitate nutrient assimilation [2]. Over the course of human history, various vegetables have been consumed in both raw and cooked forms. Early investigations indicated that cooking induces only changes in phytochemical content and physical modifications [3]. Notably, vegetable juice represents a complex multicomponent system characterized by inherently heterogeneous structural assemblies rather than discrete single compounds [4]. Among these, plant-derived EVs (PEVs) are key mediators of the bioactivity of vegetable juices [5]; however, the consequences of boiling on the nanoscale structure and function of PEVs remain poorly elucidated. Thermal processing of plant matrices is known to elicit profound biochemical transformations [6,7]. Yet, whether similar structural remodeling occurs within the architectures of PEVs during such processing remains an open question.

Ginger (*Zingiber officinale*) holds a prominent place in global culinary and medicinal traditions, consumed extensively both raw and cooked for over 5,000 years [8]. A growing body of clinical and preclinical studies has investigated the therapeutic potential of ginger supplementation, ginger-derived extracts, and ginger-derived EVs (GEVs), a subset of PEVs, in the management of diseases such as inflammatory bowel disease (IBD) [9–11]. Prior research contrasting raw versus processed ginger has revealed substantial differences in the composition and bio-efficacy of its bioactive molecules [11,12]. GEVs mediate intercellular communication through cargo transfer or membrane contact. As nanoscale components derived from ginger cells, GEVs may similarly undergo heat-induced alterations. However, whether thermal processing drives their structural reorganization and how such changes affect their functional performance remain unknown.

A key factor for EV therapeutic efficacy is their cellular uptake dynamics, which vary across EV subtypes and recipient cells [9,13,14]. The surface of EVs incorporates proteins and lipids derived from their parental cells. Following isolation, EVs can subsequently acquire a dynamic protein corona from the extracellular environment, which collectively shapes their biological function, cellular uptake, and in vivo distribution [15,16]. Therapeutic hypothermia induces temperature-dependent enrichment of apolipoprotein C1 in the protein corona of the nanocarrier surface, facilitating receptor-mediated targeting capability [17]. Biological systems frequently exhibit stimulus-responsive structural reorganization in aqueous environments [18,19]. Inspired by these findings, we hypothesized that the common thermal process of boiling could drive structural reorganization of GEVs, thereby enhancing their cellular uptake and therapeutic efficacy.

In this study, we discovered that boiling triggers nanoscale reconstitution of GEVs within ginger juice, yielding thermally reassembled EVs termed T-GEVs that feature a protein-rich shell incorporating bioactive components and metal ions from the ginger matrix. T-GEVs enhance internalization by intestinal epithelial and hepatic cells via clathrin-mediated endocytosis (CME) and alter their in vivo biodistribution. Notably, T-GEVs exhibit greater anti-inflammatory efficacy than native vesicles. Beyond their standalone activity, T-GEVs serve as an efficient oral delivery platform. Loading tumor necrosis factor-α (TNF-α) small interfering RNA (siRNA) creates a synergistic therapy. The T-GEVs temper inflammatory signaling pathways, while the siRNA directly silences the key pro-inflammatory cytokine TNF-α.

## Results

### Liver and colon show preference for T-GEVs in vivo

**Fig. 1.**
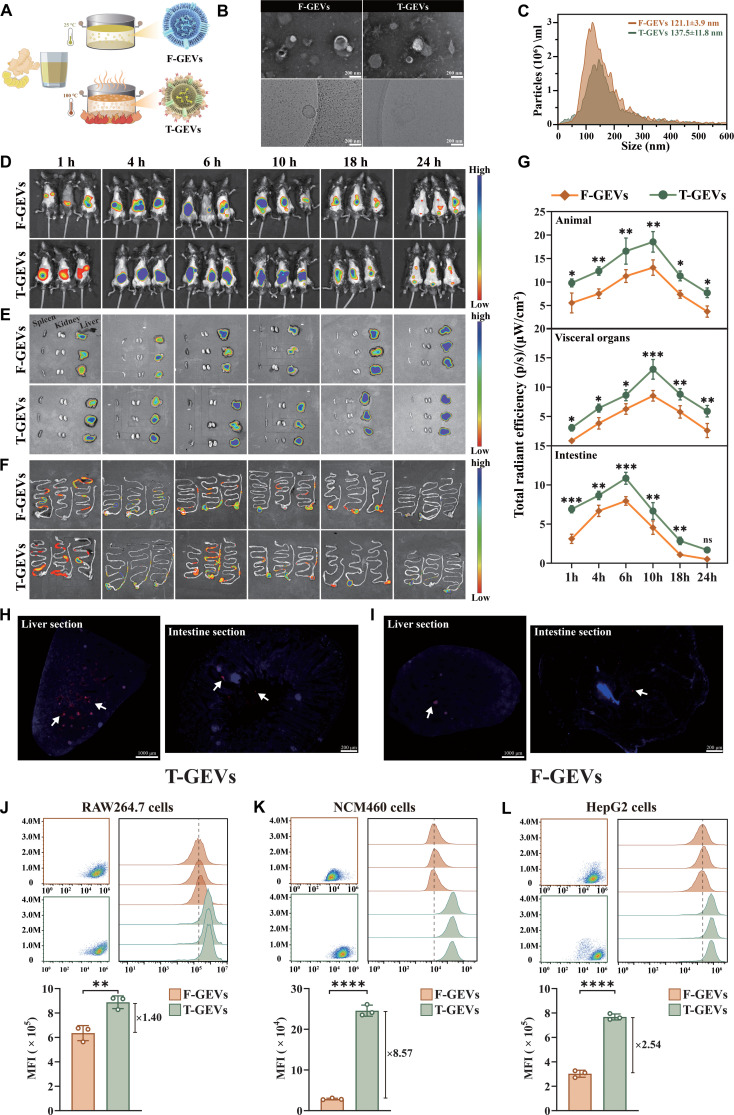
Targeted distribution and cellular uptake of T-GEVs. (A) Schematic representation of the thermally processed GEVs. (B) TEM (upper panel) and cryo-TEM (lower panel) imaging of F-GEVs and T-GEVs. (C) Size distribution analysis of F-GEVs and T-GEVs. *n* = 3. (D) Fluorescence intensity distribution in mice following oral administration of F-GEVs and T-GEVs. (E and F) Ex vivo organ imaging (spleen, kidney, liver, and intestine) at various time points post-administration. (G) Quantification of liver and colon fluorescence signals (mean ± SD, 2-tailed *t* test). *n* = 3 biologically independent samples. *P < 0.05, **P < 0.01, ***P < 0.001. (H and I) Confocal microscopic imaging of intestine and liver sections following oral administration of F-GEVs and T-GEVs. (J to L) Flow cytometric analysis of the F-GEV and T-GEV cellular internalization by RAW264.7 cells (J), NCM460 cells (K), and HepG2 cells (L). *n* = 3 biological replicates. Data are presented as means ± SD. *n* = 3. *P* values were calculated using 2-tailed *t* tests. **P < 0.01, ****P < 0.0001, ns, nonsignificant.

EVs represent critical functional components of vegetable juices, acting as pivotal mediators of interspecies communication through the transfer of bioactive molecules across biological systems [4,10,20]. To elucidate the effects of boiling processing on the biodistribution patterns of GEVs, we isolated EVs from fresh (F-GEVs) and thermally treated (T-GEVs) ginger juice (Fig. 1A). Transmission electron microscopy (TEM) revealed both F-GEVs and T-GEVs to display characteristic spherical morphologies consistent with previously described GEV features [9,21] (Fig. 1B). The cryo-TEM image shows T-GEVs in thermally processed ginger juice with a typical spherical morphology and an intact bilayer membrane, suggesting that the EVs remain intact (Fig. 1B). Consistently, nanoparticle tracking analysis (NTA) demonstrated a 14-nm increase in average particle diameter in T-GEVs relative to F-GEVs (Fig. 1C), indicating heat-induced surface modifications. Given the selective organotropism of PEVs and their therapeutic potential [21–23], we evaluated the effects of boiling on the in vivo GEV biodistribution using 1,1′-dioctadecyl-3,3,3′,3′-tetramethylindotricarbocyanine iodide (DiR)-labeled vesicles administered orally to mice. Quantitative imaging revealed that T-GEVs exhibited markedly enhanced hepatic and colonic accumulation, with 2.0- and 2.6-fold greater localization in the liver and colon, respectively, at 18 h post-administration, compared to F-GEVs (Fig. 1D to G). To further visualize the tissue distribution of F-GEVs and T-GEVs, fluorescence imaging was performed on organs isolated following oral administration. We found that T-GEVs accumulated more substantially in the intestine and liver compared to F-GEVs (Fig. 1H and I).

To assess whether enhanced organ targeting correlated with improved cellular uptake, we quantified the internalization of T-GEVs in macrophages, hepatocytes, and intestinal epithelial cells. Viability assays confirmed no cytotoxicity at 5 × 10^9^ particles/ml for either T-GEVs or F-GEVs (Fig. S1). Uptake analyses revealed that T-GEVs exhibited 8.57-, 2.54-, and 1.40-fold higher internalization in NCM460 intestinal epithelial cells, HepG2 hepatocytes, and RAW264.7 macrophages, respectively, relative to F-GEVs (Fig. 1J to L and Fig. S2). Confocal microscopy further confirmed the superior internalization of T-GEVs in RAW264.7 cells (Fig. S3). Collectively, the results suggested that T-GEVs exhibit favorable uptake profiles within the liver and intestinal microenvironment, as evidenced by increased internalization by phagocytes, hepatocytes, and intestinal epithelial cells.

### Boiling processing induces structural and compositional changes in GEVs

**Fig. 2.**
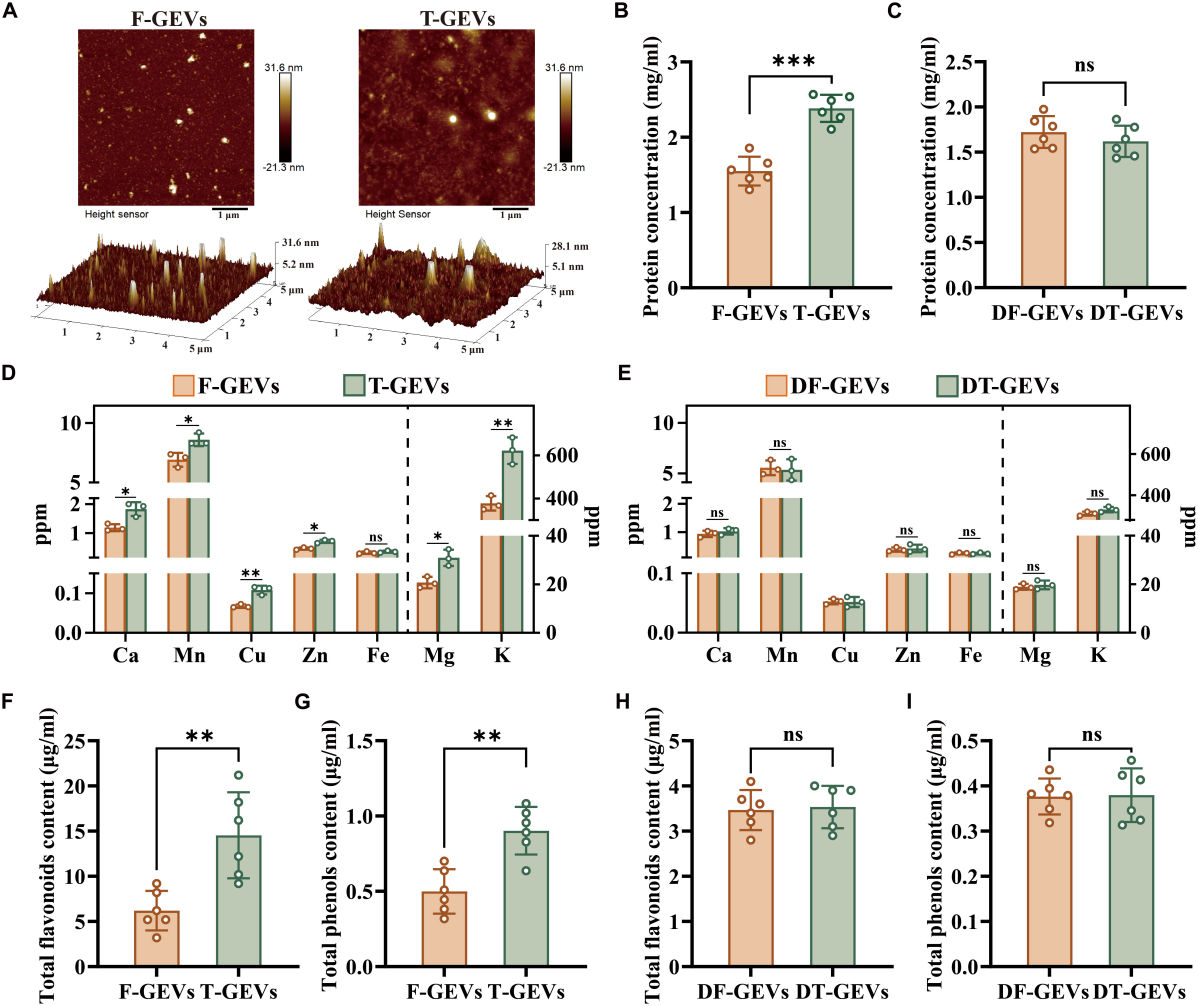
Boiling processing alters the structure and composition of GEVs. (A) AFM topography maps of F-GEVs and T-GEVs. (B and C) Protein quantification of F-GEVs and T-GEVs (B), as well as their digested forms DF-GEVs and DT-GEVs (C) by BCA assay. *n* = 3 replicates. The 2-sided unpaired *t* test method was used for statistical significance testing. ***P < 0.001, ns, nonsignificant. (D and E) ICP-MS analysis of physiological metal elements (Ca, Mn, Cu, Zn, Fe, Mg, and K) in both F-GEVs and T-GEVs (D), as well as DF-GEVs and DT-GEVs (E). *n* = 3 replicates. The 2-sided unpaired *t* test method was used for statistical significance testing. *P < 0.05, **P < 0.01, ns, nonsignificant. (F to I) Measurement of total flavonoid and total phenol content in both F-GEVs and T-GEVs (F and G) as well as DF-GEVs and DT-GEVs (H and I) by UV–visible spectrophotometry. *n* = 3 replicates. The 2-sided unpaired *t* test method was used for statistical significance testing. **P < 0.01, ns, nonsignificant.

Biological systems assemble organic–inorganic complexes in response to thermal stimuli [24]. Atomic force microscopy (AFM) revealed that T-GEVs displayed markedly increased surface roughness and heterogeneity compared to F-GEVs (Fig. 2A). Particle counts showed significantly more F-GEVs (1.3 × 10^11^ particles/ml) than T-GEVs (7.4 × 10^10^ particles/ml) (Fig. S4). Notably, protein content measurements demonstrated a 41% elevation in T-GEVs (2,336.63 ± 230.77 ng/μl) relative to F-GEVs (1,660.17 ± 193.93 ng/μl; Fig. 2B). Inductively coupled plasma mass spectrometry (ICP-MS) analysis revealed significantly increased levels of calcium (1.54-fold), manganese (1.24-fold), magnesium (1.49-fold), copper (1.61-fold), zinc (1.54-fold), and potassium (1.65-fold) in T-GEVs compared to F-GEVs (Fig. 2D). Boiling also led to a reduction in microRNA (miRNA) content within GEVs relative to the raw preparation (Fig. S5).

To assess the origin of surface-bound proteins and ions, we performed protease digestion of surface proteins on both F-GEVs and T-GEVs to yield protease-digested F-GEVs (DF-GEVs) and protease-digested T-GEVs (DT-GEVs). Strikingly, protein and metal ion levels in DT-GEVs returned to those observed in DF-GEVs (Fig. 2C and E). The results imply that the increased metal ions on T-GEVs may be mainly bound to groups on ginger proteins via chelation or self-assembly, which can be transferred intercellularly to modulate biological responses [25]. Furthermore, T-GEVs retained 1.8- and 2.3-fold higher total phenolic and flavonoid content compared to F-GEVs (Fig. 2F and G), a phenomenon absent in DT-GEVs (Fig. 2H and I). These findings indicate that boiling drives protein–phytochemical co-assembly through enhanced hydrophobic interactions, creating stabilized metal complexes that boost bioactive retention.

### Protein shell formed on the surface of EVs isolated from boiling processing-treated ginger juice

**Fig. 3.**
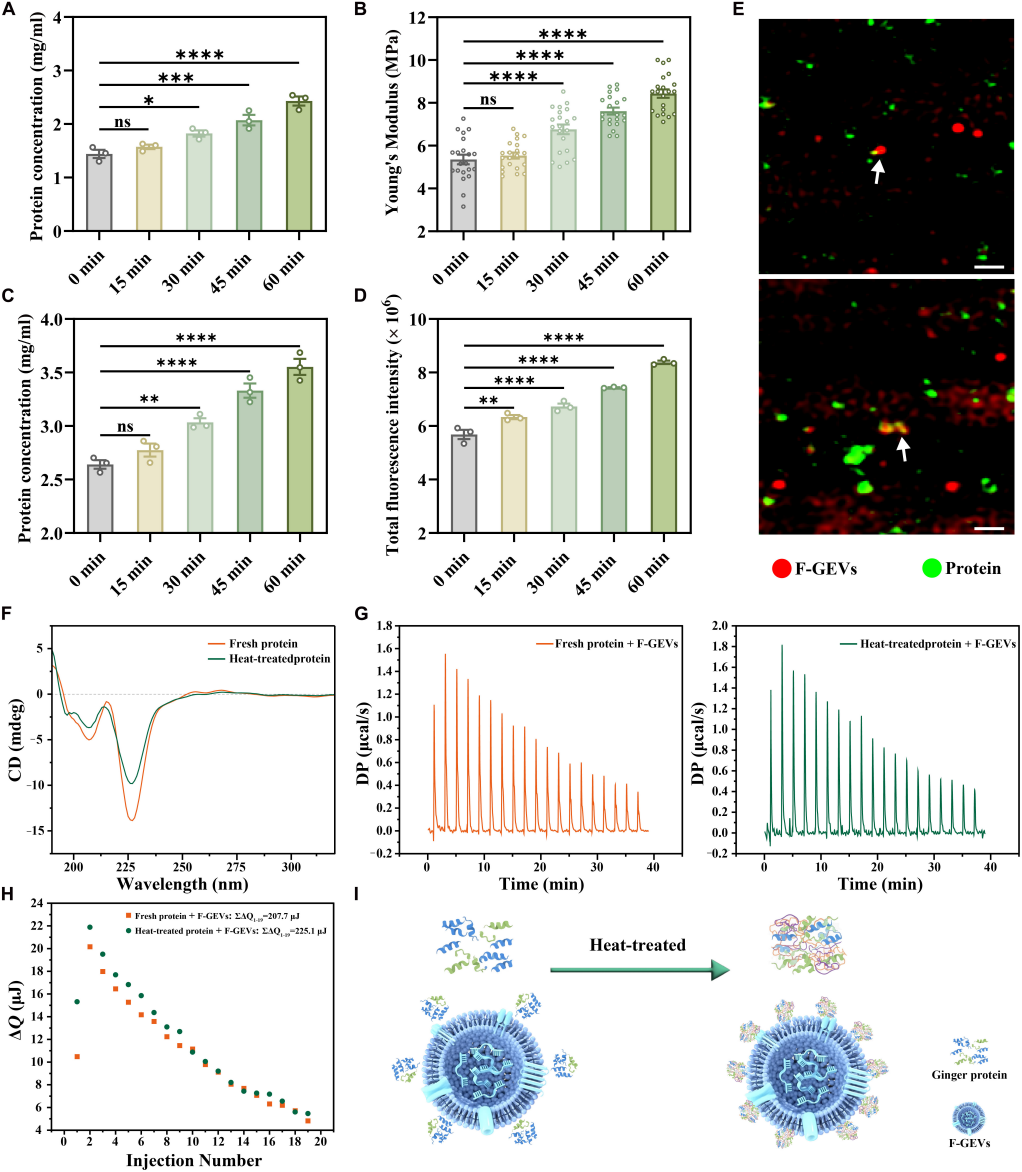
The protein shell formed on the surface of T-GEVs. (A) Time-dependent protein adsorption of T-GEVs during boiling processing (0 to 60 min). (B) AFM-measured Young’s modulus (*n* = 20 particles per group). (C) Protein concentrations after incubation of F-GEVs with fluorescently labeled proteins under controlled heating. (D) Total fluorescence value of T-GEVs at various thermal treatment time points. (E) Confocal microscopy of Alexa Fluor 488-labeled protein (green) colocalizing with DiD-labeled GEVs (red). Scale bars, 500 nm. (F) CD spectra of fresh and boiled ginger proteins. (G) ITC thermograms of fresh (left) and boiled (right) ginger proteins titrated into an F-GEV suspension. (H) Cumulative interaction heat. (I) Schematic illustration of ginger protein organization around F-GEVs before and after boiling. All data are presented as means ± SD. *n* = 3. *P* values were calculated using 2-sided one-way ANOVA post-Dunnett’s test; ***P < 0.001, ****P < 0.0001, ns, nonsignificant.

To determine whether boiling induces protein adsorption onto GEVs, we compared surface-deproteinized samples of T-GEVs and F-GEVs, which exhibited comparable levels of residual protein (Fig. 2C). This finding indicates that the higher protein content observed in T-GEVs stems from surface-adsorbed proteins, not from intrinsic compositional differences. Time-course analysis demonstrated that extended boiling progressively increased both protein content and mechanical rigidity of GEVs (Fig. 3A and B), with T-GEVs exhibiting a 1.85-fold higher Young’s modulus after 60 min of thermal treatment (7.86 ± 1.04 MPa) compared to F-GEVs (4.25 ± 1.92 MPa) (Fig. 3B). Fluorescence quantification confirmed time-dependent accumulation of surface-adsorbed proteins (Fig. 3C and D). Direct visualization using fluorescently labeled proteins incubated with GEVs under thermal conditions revealed the formation of a distinct protein shell on T-GEV surfaces (Fig. 3E). Thermal stress induced greater changes in the secondary structure of proteins in solution. Circular dichroism (CD) spectra of ginger protein at various temperatures indicate that the protein is also primarily composed of α-helices (208 and 222 nm) and that the secondary structure is gradually disrupted as the temperature is increased (Fig. 3F). Upon heating, the β-sheet ratio of ginger protein was lower. Furthermore, spectral changes in the 190- to 200-nm region upon boiling suggested the formation of non-native, partially ordered intermediates or aggregates, rather than a complete transition to a random-coil conformation (Fig. 3F). To further investigate the interaction between structural changes of ginger protein and GEVs, the thermodynamic response of protein adsorption on GEV surfaces was determined using isothermal titration calorimetry (ITC). Titration thermograms showed a markedly stronger exothermic response for boiled proteins than for their native counterparts (Fig. 3G). The cumulative interaction heat (ΣΔQ₁_–_₁₉) increased from 207.7 μJ to 225.1 μJ after thermal treatment, indicating a more favorable enthalpic drive for assembly (Fig. 3H). Collectively, these results demonstrate that thermally unfolded proteins promote energetically favorable associations with the F-GEV lipid bilayer, culminating in the formation of a stabilized protein shell on GEVs (Fig. 3I).

### Boiling-induced remodeling of the GEV proteome

**Fig. 4.**
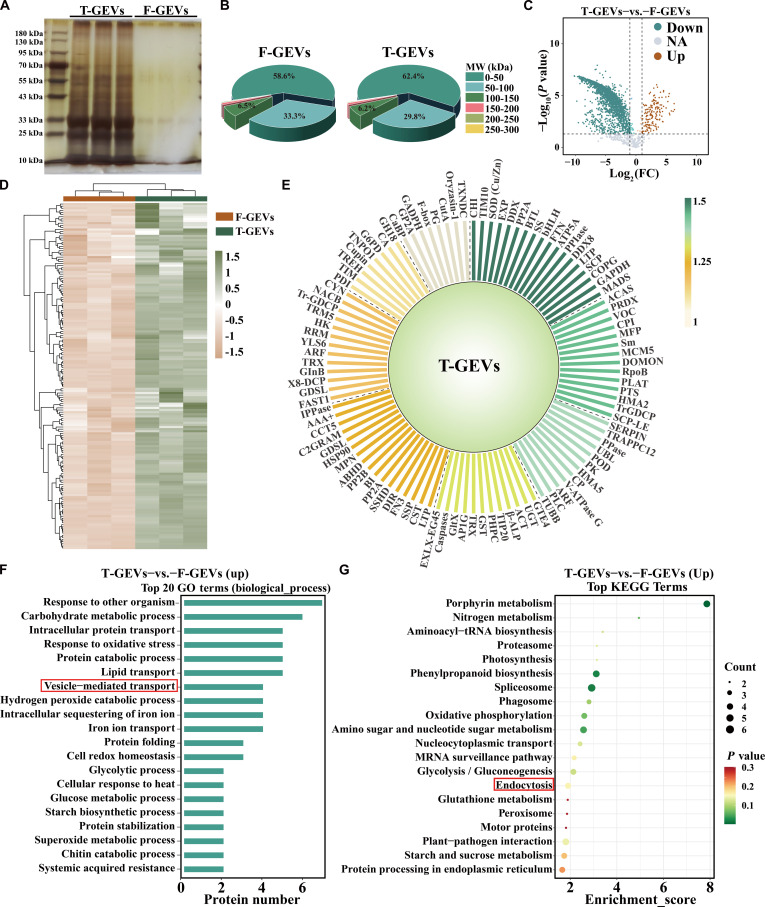
The formation of protein shell on T-GEVs was evaluated by SDS–PAGE and LC-MS/MS. (A) Silver-stained gel comparing protein profiles of F-GEVs and T-GEVs. (B) Protein molecular weight ratio in F-GEVs and T-GEVs. (C) Volcano plot of differentially expressed proteins (|log_2_FC| > 1, *P* < 0.05). (D) Hierarchical clustering of proteomic profiles. (E) Schematic representation of up-regulated T-GEV proteins with annotated functional domains. (F) GO biological process enrichment analysis of differential proteins. (G) KEGG pathway mapping of protein functional networks.

To investigate boiling processing-driven proteomic remodeling in GEVs, quantitative proteomics via liquid chromatography–MS/MS (LC-MS/MS) revealed substantial differences in protein composition between F-GEVs and T-GEVs (Fig. 4). Sodium dodecyl sulfate (SDS)–polyacrylamide gel electrophoresis (PAGE) demonstrated altered protein abundance in T-GEVs (Fig. 4A), with molecular weight distributions predominantly spanning 0 to 50 kDa (Fig. 4B). Lower-molecular-weight proteins, owing to higher diffusion coefficients, may preferentially bind T-GEV surfaces. LC-MS/MS results showed minimal mass error and strong correlation among replicates, confirming data reliability (Fig. S6A and B). In total, 2,706 proteins were identified in F-GEVs and 1,010 in T-GEVs, with no proteins uniquely detected in T-GEVs (Fig. S7), suggesting proteome reorganization rather than the acquisition of novel proteins. Volcano plot and clustering analyses identified 163 proteins significantly up-regulated in T-GEVs (Fig. 4C and D), including key regulators of vesicle transport such as V-type proton adenosine triphosphatase (ATPase) subunit G, adenosine diphosphate-ribosylation factor 1 (ARF1), and β-adaptin-like protein (Fig. 4E). Notably, thermal processing significantly increased the abundance of mannose-specific lectin-like proteins on the surface of T-GEVs (Table S1). Given their high selectivity for mannose and the prevalence of mannosylated sites on intestinal cells [26], this finding suggests that T-GEVs may possess the potential to target intestinal epithelial cells. Additional proteins included those involved in immunity, metabolism, signaling, chaperone function, and protein storage (Table S1). Gene Ontology (GO) and Kyoto Encyclopedia of Genes and Genomes (KEGG) enrichment analyses further indicated that up-regulated proteins in T-GEVs were enriched in pathways associated with molecular regulation, protein transport, vesicle-mediated transport, and endocytic processes (Fig. 4F and G), supporting the hypothesis that boiling-driven proteomic remodeling enhances vesicle internalization capacity.

### Boiling processing facilitates the incorporation of CME signaling proteins into GEVs

**Fig. 5.**
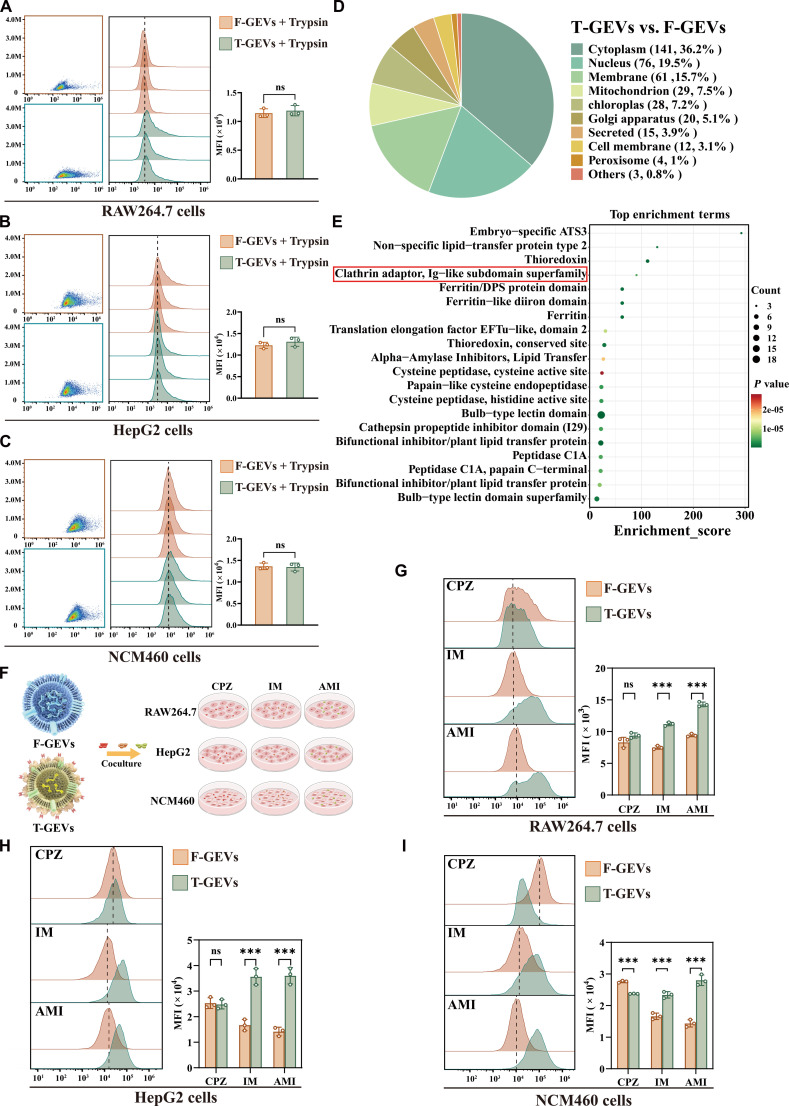
Internalization of T-GEVs is controlled by the clathrin-related endocytosis machinery. (A to C) Uptake of DF-GEVs and DT-GEVs in RAW264.7 cells (A), HepG2 cells (B), and NCM460 cells (C). *n* = 3 biologically independent samples. The 2-sided unpaired *t* test method was used for statistical significance testing. ns, nonsignificant. (D) Subcellular localization analysis. (E) InterPro analysis. (F) Experimental design. (G to I) Uptake of F-GEVs and T-GEVs in RAW264.7 cells (G), HepG2 cells (H), and NCM460 cells (I), with chlorpromazine (CPZ), indomethacin (IM), and amiloride (AMI). *n* = 3. The 2-sided unpaired *t* test method was used for statistical significance testing. ***P < 0.001, ns, nonsignificant.

Uptake assays, both in vitro and in vivo, demonstrated the crucial role of the T-GEV protein shell in regulating cellular uptake. To further confirm the contribution of the protein shell, the cellular uptake of DT-GEVs and DF-GEVs was assessed. As shown in Fig. 5A to C, DT-GEV uptake levels were comparable to DF-GEVs in RAW264.7, NCM460, and HepG2 cells, supporting the role of the protein shell in mediating endocytosis. It is well established that the formation of a biomolecular corona on the surface of nanoparticles can obscure their target proteins, thereby reducing targeting efficiency [16]. However, despite the presence of a protein shell, T-GEVs maintain their targeting function. This enhanced targeting capability is likely attributed to the unique composition of the protein shell on T-GEVs. Subcellular localization analysis revealed that the up-regulated proteins in T-GEVs predominantly originated from the plasma membrane, cytoplasm, and Golgi apparatus (Fig. 5D), suggesting a potential role in transmembrane transport that may facilitate the efficient intracellular delivery of T-GEVs [27]. To elucidate mechanisms underlying enhanced T-GEV uptake, we conducted functional validation through pharmacological interrogation with pathway-specific inhibitors. InterPro analyses identified up-regulation of proteins associated with clathrin-mediated endocytosis (CME) (Fig. 5E). An inhibitor targeting the CME pathway was utilized in the treatment of both nonphagocytic and phagocytic cells (Fig. 5F). The addition of chlorpromazine (CPZ), a known inhibitor of CME [10], remarkably decreased the cellular uptake efficiencies of T-GEVs in RAW264.7, NCM460, and HepG2 cells. Notably, a comparable internalization efficiency was observed within a 12-h period following the uptake of both T-GEVs and F-GEVs in RAW264.7 (Fig. 5G) and HepG2 cells (Fig. 5H). Moreover, the administration of CPZ resulted in a reduction in the cellular uptake efficiencies of T-GEVs compared to F-GEVs in the NCM460 cells (Fig. 5I). Given the limited specificity of individual inhibitors, we utilized multiple compounds targeting distinct steps of the CME pathway—namely, Pitstop 2, ES9-17, and Dynole 2-24 (Fig. S8). The internalization of T-GEVs was consistently reduced by each inhibitor, supporting the involvement of CME in cellular uptake. Notably, despite inhibiting macropinocytosis and caveolin-mediated phagocytosis pathways, the internalization of T-GEVs was significantly enhanced (Fig. 5G to I). After treatment with F-GEVs or T-GEVs, the levels of membrane-associated clathrin showed no significant increase in RAW264.7, NCM460, or HepG2 cells (Fig. S9). Overall, these findings confirm that boiling promotes the incorporation of CME-related proteins into GEVs, thereby enhancing their cellular internalization across phagocytic and nonphagocytic cells.

### T-GEVs possess intrinsic anti-inflammatory activity as a therapeutic agent

**Fig. 6.**
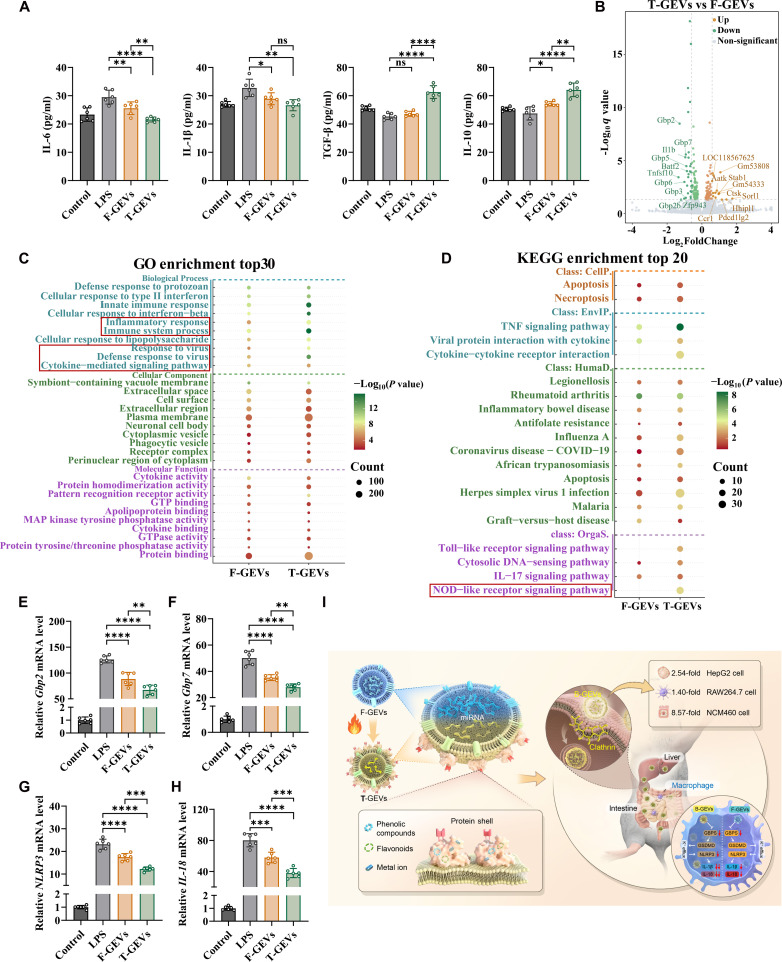
Superior anti-inflammatory performance of T-GEVs over F-GEVs. (A) ELISA analysis showing the levels of inflammatory cytokines and TGF-β in macrophages transfected with F-GEVs and T-GEVs. (B) Volcano plot showing differentially expressed genes between F-GEVs and T-GEVs. (C) GO enrichment analysis in F-GEV- and T-GEV-treated macrophages. (D) KEGG pathway analysis. (E to H) Expression of key genes in the NOD-like receptor signaling pathway after the addition of F-GEVs and T-GEVs, including *Gbp2* (E), *Gbp7* (F), *NLRP3* (G), and *IL-18* (H). (I) Schematic illustrating the large-scale generation of functional T-GEVs from boiled ginger juice and their mechanism of anti-inflammatory action. All data are presented as means ± SD. *n* = 6. *P* values were calculated using 2-sided one-way ANOVA post-Dunnett’s test; *P < 0.05, **P < 0.01, ***P < 0.001, ****P < 0.0001.

The distinct structural and compositional features of T-GEVs compared to F-GEVs likely account for their divergent biological activities. In an lipopolysaccharide (LPS)-stimulated RAW264.7 macrophage model of diet-induced inflammation, T-GEVs significantly suppressed pro-inflammatory cytokines, increased anti-inflammatory interleukin-10 (IL-10), and reduced transforming growth factor-β (TGF-β) secretion, unlike F-GEVs (Fig. 6A and Fig. S10). No significant differences were observed in TNF-α, IL-1β, TGF-β, and IL-6 expression between DT-GEV and DF-GEV treatment groups (Fig. S11).

RNA-sequencing (RNA-seq) analysis was performed on RAW264.7 cells treated with F-GEVs or T-GEVs to explore the transcriptomic changes induced by boiling. RNA-seq revealed distinct transcriptional profiles (Figs. S12 and S13), with 46 differentially expressed genes (*P* < 0.05, |log_2_FC| > 1) between F-GEV- and T-GEV-treated macrophages (Fig. S14). Down-regulation of Gbp family genes was associated with restrained inflammatory activation and a more balanced antiviral response [28]. T-GEV treatment significantly down-regulated key inflammatory genes (i.e., *Gbp2*, *Gbp5*, *Gbp7*, *Tlr3*, and *Ifit1*) and up-regulated genes associated with endocytic recycling (*Sorl1* and *Stab1*) (Fig. 6B). These transcriptional changes suggest that T-GEVs promote an anti-inflammatory and antiviral cellular response. The primary down-regulated biological process category involves terms like “innate immune response”, “inflammatory response”, “cellular response to interferon-beta adhesion”, “immune system process”, and “defence response to virus” (Fig. 6C). Notably, GO and KEGG analysis of the up-regulated genes in T-GEV-treated cells indicated enrichment of pathways involved in endocytic recycling, vesicular transport, and low-density lipoprotein (LDL) particle binding. These bioinformatic findings are consistent with our prior results (Fig. 4F and G) and further suggest a potential role for T-GEVs in promoting endocytic processes and facilitating active EV uptake (Figs. S15 and S16). Analyses highlighted pathways linked to antiviral responses and inflammation, including nucleotide-binding oligomerization domain (NOD)-like receptor, Toll-like receptor, and IL-17 signaling pathways (Fig. 6D). Quantitative real-time polymerase chain reaction (RT-qPCR) validation confirmed reduced expression of *Gbp2*, *Gbp7*, *NLRP3*, and *IL-18* in T-GEV-treated LPS-stimulated RAW264.7 macrophage (Fig. 6E to H), supporting the conclusion that T-GEVs exert superior anti-inflammatory effects through modulation of Gbp family genes and suppression of NLRP3 inflammasome activation. Our study demonstrates that boiling facilitates the generation of bioactive T-GEVs (Fig. 6I), offering a novel approach to natural engineering.

### T-GEVs as a synergistic therapeutic platform combining innate anti-inflammatory activity with efficient siRNA delivery

**Fig. 7.**
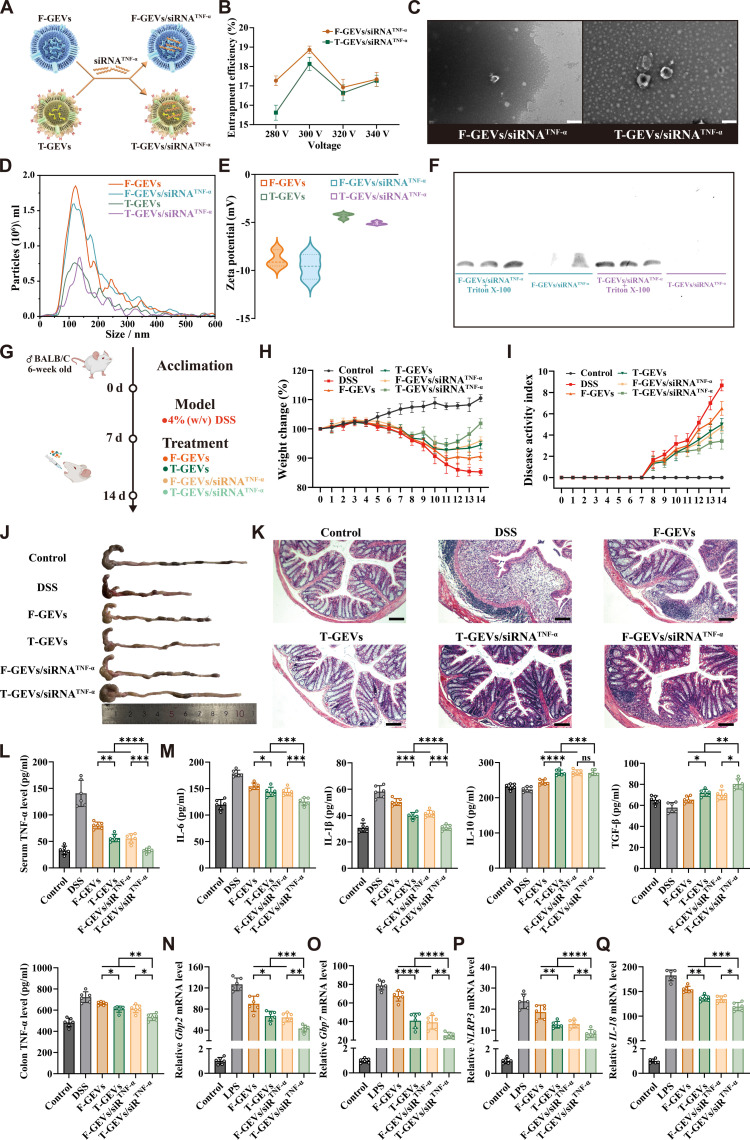
Therapeutic effects of T-GEVs/siRNA^TNF-α^ in IBD models. (A) Schematic illustration of the preparation of F-GEVs/siRNA^TNF-α^ and T-GEVs/siRNA^TNF-α^. (B) Loading efficiency of TNF-α siRNA into F-GEVs and T-GEVs at different electroporation voltages. (C) Representative TEM photos of F-GEVs/siRNA^TNF-α^ and T-GEVs/siRNA^TNF-α^. Scale bar, 200 nm. (D and E) Particle size (D) and zeta potential (E). (F) Ribonuclease protection assay assessing siRNA stability. (G) Experimental scheme for DSS-induced acute colitis and therapeutic administration. (H) Body weight changes in each group on day 14. (I) DAI score. (J) Representative photographs of excised colon tissues. (K) Analysis of colon tissues using H&E assays post-treatment. Scale bars, 100 μm. (L) TNF-α level of serum and colon tissue after treatment of colitis. (M) ELISA-based measurement of inflammatory cytokines and TGF-β levels in colon tissue. (N to Q) Relative mRNA expression levels of *Gbp2* (N), *Gbp7* (O), *NLRP3* (P), and *IL-18* (Q) were measured by RT-qPCR. All data are presented as means ± SD. *n* = 6. P values were calculated using 2-sided one-way ANOVA post-Dunnett’s test; *P < 0.05, **P < 0.01, ***P < 0.001, ****P < 0.0001, ns, nonsignificant.

Given the established intrinsic anti-inflammatory properties and efficient intestinal absorption of T-GEVs, we explored their potential as an all-in-one synergistic therapy. We hypothesized that combining the innate immunomodulatory activity of T-GEVs with targeted gene silencing would improve therapeutic outcomes. To this end, we developed an oral siRNA delivery system using T-GEVs (Fig. 7A). The TNF-α siRNA was loaded into both F-GEVs and T-GEVs using optimized electroporation parameters (300 V), achieving a loading efficiency of approximately 18% (Fig. 7B and Fig. S17). TEM revealed that both T-GEVs/siRNA^TNF-α^ and F-GEVs/siRNA^TNF-α^ retained their intact structures, exhibiting a typical cup-shaped morphology with nanoscale dimensions (Fig. 7C). Following siRNA loading, the hydrodynamic diameter increased to 146.3 ± 8.3 nm for T-GEVs/siRNA^TNF-α^ and 133.5 ± 4.5 nm for F-GEVs/siRNA^TNF-α^, while the surface potential decreased to −5.07 ± 0.77 mV and −9.70 ± 0.85 mV, respectively (Fig. 7D and E). Gel electrophoresis further demonstrated that the nanovesicle membranes effectively protected the encapsulated siRNA from degradation unless chemically lysed (Fig. 7F).

We directly compared the therapeutic efficacy of T-GEVs/siRNA^TNF-α^, F-GEVs/siRNA^TNF-α^, F-GEVs alone, and T-GEVs alone in a dextran sodium sulfate (DSS)-induced colitis model. Mice receiving 4% DSS in drinking water began losing weight on day 5 due to severe diarrhea and reduced food intake (Fig. 7G). After 6 d of treatment, the proportion of weight loss alleviated and the disease activity index (DAI) decreased following treatment with T-GEVs/siRNA^TNF-α^ or F-GEVs/siRNA^TNF-α^ compared to the DSS, F-GEV, or T-GEV groups (Fig. 7H and I). Although both F-GEV and T-GEV treatments provided benefits, T-GEVs alone offered significantly better protection against body weight loss and colon shortening than did F-GEVs (Fig. 7H and J), reaffirming their enhanced intrinsic activity. The synergistic effects of T-GEVs and TNF-α siRNA were evident in the significantly higher weight and lower DAI in the T-GEVs/siRNA^TNF-α^ group compared to the other groups (Fig. 7H and I). Treatment with T-GEVs/siRNA^TNF-α^ produced the most profound therapeutic recovery, while T-GEVs/siRNA^TNF-α^ recipients showed nearly normalizing colon length and histology compared to other groups, owing to its synergistic therapeutic effects (Fig. 7H to J). Hematoxylin and eosin (H&E) staining indicated that the T-GEVs/siRNA^TNF-α^ group exhibited the most substantial alleviation of colonic epithelial damage (Fig. 7K). Furthermore, no significant histopathological alterations were observed in the liver or kidney tissues across all treatment groups (Fig. S18). Meanwhile, oral administration of T-GEVs/siRNA^TNF-α^ led to a significant reduction in TNF-α levels in both serum and colonic tissues, outperforming the F-GEVs, T-GEVs, and F-GEVs/siRNA^TNF-α^ groups (Fig. 7L). Furthermore, T-GEVs/siRNA^TNF-α^ treatment markedly decreased the pro-inflammatory cytokines IL-6 and IL-1β while elevating TGF-β (Fig. 7M). In contrast, it substantially reduced IL-10, a potent immunosuppressive cytokine (Fig. 7M). Notably, reductions in *Gbp2*, *Gbp7*, *NLRP3*, and *IL-18* were observed specifically in the T-GEVs/siRNA^TNF-α^ group compared to the other 3 groups (Fig. 7N to Q). Together, these data suggest that oral administration of T-GEVs/siRNA^TNF-α^ can effectively hinder the development of intestinal inflammation, demonstrating significantly better therapeutic effects compared to T-GEVs or F-GEVs/siRNA^TNF-α^ therapy alone. The T-GEVs/siRNA^TNF-α^ platform exerts a synergistic, multi-layered anti-inflammatory effect by simultaneously modulating upstream regulatory pathways through its innate cargo and silencing downstream effector genes via delivered siRNA.

## Discussion

Debate persists regarding the relative health benefits of raw versus thermally processed forms [2,3], largely due to limited understanding of how different cooking methods alter the structural and functional properties of bioactive constituents. PEVs in vegetable juices form the functional basis of their bioactivity [10,11]. Yet, how a ubiquitous process such as boiling mechanistically influences PEVs’ functionality has remained unclear. Our work addresses a critical oversight by demonstrating that boiling actively reconstitutes PEVs, fundamentally reframing it from a passive, potentially degradative culinary step into an active, structure-engineering strategy that enhances their health-promoting and therapeutic potential. Here, we demonstrate that thermal processing actively reconfigured T-GEVs with a proteinaceous shell enriched in native proteins, phytochemicals, and physiological metals. This reconstituted architecture not only enhances cellular uptake and anti-inflammatory efficacy but also positions T-GEVs as potent platforms for nucleic acid therapeutics.

Comparative isolation and analysis of T-GEVs and F-GEVs revealed marked differences in particle size, rigidity, and morphology. TEM and cryo-TEM revealed typical vesicular morphology of T-GEVs and F-GEVs (Fig. 1B), akin to the recognized characteristics of vesicles derived from mammalian cells [29]. NTA indicated a moderate increase in mean diameter (∼14 nm) in T-GEVs relative to F-GEVs, which is unlikely to be the primary determinant of altered biodistribution [30]. Changes in EVs outer membrane size are associated with compositional shifts [31], offering a basis for differential biodistribution. Following oral administration, T-GEVs preferentially accumulated in intestinal and hepatic tissues. This biodistribution profile highlights their therapeutic potential for interventions targeting gut–liver axis disorders.

Because efficient cellular internalization is essential for PEVs to exert cross-kingdom effects [10,32,33], we investigated the basis for the enhanced uptake of T-GEVs. The formation of protein shells in T-GEVs was confirmed via comparative analysis of morphology, protein concentration, particle size, and rigidity relative to F-GEVs. Ginger juice is a complex system rich in multiple components, including EVs, proteins, polyphenols, and metal ions [8–12]. As in biological systems that undergo stimulus-induced structural reconfiguration in liquid environments [18,19], the protein shell in T-GEVs appears to arise from thermally driven interfacial self-assembly of ginger-derived proteins, phytochemicals, and metal ions. CD analysis further showed that heating gradually disrupts the α-helical structure of ginger proteins and reduces their β-sheet proportion; boiling induces the formation of non-native, partially ordered intermediates or aggregates, rather than a complete random-coil transition (Fig. 3F). Denatured or partially unfolded proteins can acquire new bioactivities through altered receptor engagement or membrane affinity [34]. Thermal processing also promotes the formation of protein–polyphenol assemblies [35,36]. T-GEVs retained 1.8- and 2.3-fold higher total phenolic and flavonoid content, respectively, compared to F-GEVs (Fig. 2F and G). Notably, boiling reduced the abundance of several endogenous miRNAs in T-GEVs. As PEV miRNAs have been implicated in cross-kingdom regulation of host gene expression [9,10,20], this change could potentially influence their biological activity. However, despite the reduction in endogenous miRNA cargo, T-GEVs still exhibited enhanced intestinal uptake and anti-inflammatory efficacy. These findings suggest that boiling-induced surface remodeling is likely a major contributor to the improved bioactivity observed in this study. Nevertheless, based on the current data, the precise contribution of altered miRNA cargo cannot be distinguished from that of surface remodeling and will require comprehensive small RNA profiling together with functional studies in future work. Together, these results indicate that the nanostructural reconstitution of T-GEVs through boiling represents a scalable strategy for function-oriented food engineering.

Our proteomic analyses revealed substantial remodeling of the T-GEV proteome, providing mechanistic insights into their enhanced uptake properties. Specifically, the protein shell of T-GEVs was enriched in proteins associated with vesicular trafficking and endocytosis, including ARF1 and adaptor complex components essential for clathrin-coated vesicle formation [37,38]. Given that endocytosis is essential for intracellular delivery of EV cargo [38,39], the elevated internalization of T-GEVs by phagocytes, hepatocytes, and enterocytes supports the notion that boiling-induced proteomic changes facilitate uptake. Most notably, this reconstituted surface architecture mediates a striking 8.57-fold increase in uptake efficiency specifically within intestinal epithelial cells. EV uptake generally proceeds via macropinocytosis, phagocytosis, and clathrin-/caveolin-mediated endocytosis [40]. Engineered EVs carrying fusion proteins can facilitate targeted uptake by recipient cells [41,42]. Our proteomic data indicated an up-regulation of proteins related to transport and CME, suggesting that enhanced uptake of T-GEVs is CME-dependent. Uptake assays, both in vivo and in vitro*,* demonstrated the crucial role of the T-GEV protein shell in regulating uptake-related events. The observed enrichment of CME-related proteins in T-GEVs supports these findings. The surface of T-GEVs thus appears to actively engage the host cell’s endocytic machinery—a feature typically engineered into synthetic nanoparticles but here emerging from simple thermal processing.

Ginger and its extracts have a long history of medicinal use, particularly in alleviating gastrointestinal disorders [8–11]. We investigated whether boiling—a common culinary practice—could enhance the bioactivity of GEVs. Previous studies have established that edible GEVs exhibit immunomodulatory properties [9,43]. Our findings demonstrate that thermal processing substantially enhances the anti-inflammatory functionality of GEVs, suggesting practical applications for T-GEVs as bioactive components in dietary interventions. Specifically, we found that T-GEVs exert a pronounced inhibitory effect on the NLRP3 inflammasome, which is a central mediator of inflammatory pathology. Mechanistically, this suppression is linked to their ability to modulate the expression of Gbp family genes, a class of interferon-inducible guanosine triphosphatases that critically regulate NOD-like receptor signaling and inflammasome activation [44]. By attenuating this key inflammatory cascade, T-GEVs effectively dampen NOD-like receptor-mediated pro-inflammatory signaling. In a DSS-induced colitis model, T-GEVs effectively attenuated inflammatory signaling, demonstrating that a simple thermal step can transform dietary EVs into potent, physiologically relevant immunomodulators.

We developed T-GEVs into an oral siRNA delivery platform by exploiting their enhanced intestinal uptake and intrinsic tissue tropism. Plant-derived nanoparticle systems have been used for oral siRNA delivery in colitis models, demonstrating the feasibility of siRNA transport [45,46]. However, limitations still exist for oral siRNA delivery using PEVs in colitis. Although modifying the surface proteins of PEVs can mitigate off-target toxicity to normal tissues [47], such strategies often introduce additional complexity and manufacturing hurdles. Building on this foundation, our work employs a simple heat-induced reconstitution of T-GEVs to integrate inherent bioactive components with targeted intestinal siRNA delivery, thereby overcoming the abovementioned problems. Our work redefines T-GEVs into a programmable, synergistic therapeutic platform. This establishes a dual-modal therapeutic strategy that combines endogenous immunomodulatory activity with gene silencing. In IBD, where single-target therapies often show limited efficacy [48], T-GEVs/siRNA^TNF-α^ embodies an integrated synergistic strategy. The native components of T-GEVs exert broad immunomodulatory effects, while the siRNA payload directly and specifically silences a key pro-inflammatory mediator, TNF-α. It also suggests that the platform’s utility can be extended by loading different siRNA cargos to target other disease-specific genes, all while leveraging the inherent immunomodulation activity and intestinal tropism of the T-GEV scaffold.

Notably, this thermally induced reassembly platform offers advantages over existing PEV engineering strategies. Genetic engineering of plants to produce EVs is expensive [49], while chemical modification risks unintended alterations and side effects [50]. T-GEVs provide unique morphological and compositional features with low immunological risk and attractive cost-effectiveness at large scale. Recent work showed that half-dose 5-aminosalicylic acid (5-ASA) combined with *Lactiplantibacillus plantarum*-derived EVs (LP-EVs) is non-inferior to full-dose 5-ASA in alleviating colitis symptoms, suggesting a strategy to reduce the 5-ASA dosage required for ulcerative colitis (UC) therapy [51]. Similarly, T-GEVs exhibit potent anti-inflammatory effects, and their combination with 5-ASA enhances efficacy while reducing both dosage and dosing frequency. Together, these findings support an emerging paradigm of using dietary or probiotic-derived EVs to optimize conventional pharmacotherapy for IBD. For IBD, oral T-GEVs act directly on the intestinal mucosa, enabling local therapy with minimal systemic side effects via synergistic anti-inflammatory and gene-silencing activities. In contrast, liver diseases pose greater challenges, including Kupffer cell uptake and the sinusoidal barrier after portal vein entry [52]. Leveraging their oral stability, intrinsic anti-inflammatory properties, and targeting capability, T-GEVs can deliver drugs such as 5-ASA and budesonide directly to intestinal lesions, increasing local drug concentrations while providing synergistic anti-inflammatory effects. Moreover, the high potency of nucleic acid therapeutics can partially compensate for the low loading capacity of conventional delivery systems, as effective gene silencing requires only low doses [53].

Unavoidably, certain limitations exist in our work. The DSS-induced acute colitis model, the standard preclinical IBD model, primarily mimics ulcerative colitis features [11] but lacks Crohn’s disease hallmarks such as transmural inflammation, granuloma formation, and chronic relapsing progression [54]. Therefore, T-GEV efficacy requires validation in Crohn’s disease-like models, such as 2,4,6-trinitrobenzenesulfonic acid (TNBS)-induced colitis, to confirm their clinical translational potential.

## Materials and Methods

### Materials

The BCA (bicinchoninic acid) protein assay kit, LPS, 3,3′-dioctadecyloxacarbocyanine perchlorate (DiO), and 6-indolecarbamidine dihydrochloride (DAPI) were procured from Shanghai Beyotime Biotechnology. The exosome protein specific lysis buffer and 1,1′-dioctadecyl-3,3,3′,3′-tetramethylindotricarbocyanine iodide (DIR) were purchased from Shanghai Nonin Biological Technology Co. Ltd. Enzyme-linked immunosorbent assay (ELISA) kits were supplied by Shanghai Youxuan Biotechnology Co. Ltd. Fetal bovine serum (FBS), amiloride, indomethacin, and CPZ were supplied by Merck & Co. Inc., while Dulbecco’s modified Eagle’s medium (DMEM) was provided by Thermo Fisher Scientific Inc. The Cell Counting Kit-8 (CCK-8) was purchased from Hefei Biosharp. Penicillin and streptomycin were obtained from Invitrogen. All other chemicals used were of analytical grade.

### Isolation and purification of F-GEVs and T-GEVs

The fresh ginger rhizomes were thoroughly washed with ultrapure water, then sliced into small pieces using a knife, and placed into a blender. After being squeezed, the juice was immediately filtered through a 100-mesh sieve to remove residual fibers. Ginger juice from the same batch was divided into 2 groups: untreated sets and boiled-treated sets. The thermal treatment group was heated at 100 °C for 60 min, whereas the untreated group was maintained at 4 °C. All samples were stored at 4 °C prior to EV isolation. F-GEVs and T-GEVs were isolated using differential centrifugation and commercial exosome isolation kits as previously described [13,20]. Purified EVs were kept at −80 °C for future use. Protein concentration of F-GEVs and T-GEVs was measured using a BCA protein assay kit.

### Characterization of F-GEVs and T-GEVs

The morphology of F-GEVs and T-GEVs was observed using an HT7700 TEM. Briefly, samples were placed onto 200-mesh copper grids for 5 min, negatively stained with phosphotungstic acid for 1 min, and imaged at an accelerating voltage of 100 kV. For cryo-TEM imaging, F-GEVs and T-GEVs were applied to a quantifoil holey carbon grid. Following loading into a Leica EM GP2 plunge freezer, images were collected on a TEM (Talos F200C or Glacios) operating at 200 kV. Particle counts and sizes were determined by NTA (M Particle Metrix, Meerbusch, Germany), and the zeta potential was measured using laser diffraction spectrometry (Malvern Mastersizer 2000, Malvern).

### Cytotoxicity assay

RAW264.7 and NCM460 cells were plated in 96-well plates at a density of 5 × 10^3^ cells per well and cultured for 24 h. Cells were then treated with serial dilutions of F-GEVs or T-GEVs (ranging from 0 to 5 × 10^10^ particles/ml) for 24 h. Cell viability was assessed using the CCK-8 assay, and absorbance was measured at 450 nm.

### Determination of total phenols and total flavonoids

A sample solution was prepared for total phenol and total flavonoid content analysis by homogenizing the GEV extract with 50% ethanol. For total phenolic content analysis, 40 μl of the sample was mixed with 1 ml of Folin–Ciocalteu reagent. After 5 min, 0.8 ml of 7.5% sodium carbonate was added. The mixture was incubated in the dark for 30 min, and absorbance was measured at 765 nm. For total flavonoids, 1 ml of the sample was mixed with 150 μl of 5% sodium nitrite, followed by 300 μl of 10% aluminum nitrate after 5 min, and then 1 ml of 1 M sodium hydroxide. The final volume was adjusted to 4 ml with deionized water. Absorbance was recorded at 510 nm.

### Metal analysis

The concentrations of copper, iron, zinc, manganese, magnesium, potassium, and calcium were quantified using ICP-MS. Lyophilized GEV samples were digested overnight at room temperature in 50 μl of 65% (v/v) nitric acid. After heating to 90 °C for 20 min, samples were diluted to 0.5 ml with 1% (v/v) nitric acid and analyzed using an Agilent 7900 ICP-MS with helium collision mode under standard multi-element settings.

### Dynamic simulation of protein layer formation

Ginger-derived proteins were co-incubated with F-GEVs at 100 °C. At specified time points, protein concentrations were measured using a BCA assay. Proteins were pre-labeled with Alexa Fluor 488 and incubated with F-GEVs at 100 °C for 60 min. Following incubation, F-GEVs were counterstained with 1,1′-dioctadecyl-3,3,3′,3′-tetramethylindodicarbocyanine perchlorate (DiD). After ultrafiltration to remove excess dye, DiD-labeled F-GEVs were imaged using confocal laser scanning microscopy (Olympus FV4000). Fluorescence intensity was subsequently measured using a microplate reader (SuPerMax 3200) at excitation wavelengths of 488 nm.

### Proteomics analysis of F-GEVs and T-GEVs

Protein extraction from F-GEVs and T-GEVs was conducted using Exosome Protein Specific Lysis Buffer. The subsequent proteomic data analysis was executed by Shanghai OE Biotech Co. Ltd. (Shanghai, China). LC-MS/MS was performed on a Tims TOF Pro mass spectrometer (Bruker) with an Easyspray ion source. Proteins were digested with trypsin, desalted, and analyzed using data-independent acquisition (DIA). MS/MS spectra were analyzed using Spectronaut Pulsar 18.7 (Biognosys), with searches performed against the Uniprot database (uniprot_proteome_UP000734854_Zingiber officinale_20240821.fasta).

### Rigidity measurements of F-GEVs and T-GEVs

Ten microliters of F-GEVs and T-GEVs was adsorbed onto clean mica sheets and then subjected to multiple rinses with an appropriate volume of pure water. Following the removal of excess liquid using filter paper, the mica sheets were fixed onto an iron plate. Subsequently, the differences in surface rigidity among the F-GEVs and T-GEVs were analyzed using Dimension Icon.

### Secondary structure analysis of ginger proteins pre- and post-boiling

Ginger proteins were dissolved in phosphate buffer (10 mM, pH 7.4) to a final concentration of 0.2 mg·ml^−1^. Fresh samples were analyzed directly, while boiled samples were obtained by heating the solution at 100 °C for 60 min and then rapidly cooling it to room temperature. To remove insoluble aggregates, the samples were centrifuged at 12,000*g* for 10 min, and the supernatants were collected for analysis. Far-ultraviolet (UV) CD spectra were recorded at 25 °C using a spectropolarimeter equipped with a 0.1-cm path length quartz cuvette. Data were collected from 190 to 320 nm at 100 nm/min with a 1-nm bandwidth and 1-s response time. Each reported spectrum is the average of 3 consecutive scans, corrected against a buffer baseline, and expressed as mean residue ellipticity (mdeg).

### ITC analysis

We used ITC (MicroCal PEAQ-ITC) to determine the binding affinity of F-GEVs for ginger proteins before and after boiling. GEVs (1.3 × 10^10^ particles/ml) were loaded into the sample cell, and ginger protein (2 mg/ml) was placed in the injection syringe. Titrations were carried out at 25 °C with stirring at 750 rpm. The protocol included 19 injections: an initial 0.4-μl injection followed by 18 injections of 2 μl each, with 120-s intervals. Data analysis was performed with the instrument’s dedicated software.

### SDS–PAGE

Equal numbers of particles from F-GEVs and T-GEVs were lysed and loaded onto 12% SDS–PAGE gels. Protein ladders (Precision Plus Dual Color, Bio-Rad) were used for molecular weight estimation. Gels were electrophoresed at 80 V for 30 min and then at 120 V for 60 min. Bands were visualized via silver staining and imaged using a Tanon MINI Space 3000 system.

### Cellular uptake assay

RAW264.7 cells were cultured on confocal dishes and incubated with DiR-labeled F-GEVs or T-GEVs (5 × 10^9^ particles/ml) for 4 or 12 h. After incubation, cells were washed with cold phosphate-buffered saline (PBS), fixed with 4% paraformaldehyde, and stained with DAPI and DiO. Uptake was visualized using a Leica STELLARIS5 confocal microscope. No significant difference in DiO fluorescence intensity was observed between F-GEVs and T-GEVs (Fig. S19).

To further confirm the uptake of F-GEVs or T-GEVs in vitro, a quantitative analysis via flow cytometry was conducted. The F-GEVs or T-GEVs were labeled with DiO. Utilizing a flow cytometer, fluorescence intensities (λ = 484 nm) were measured in RAW264.7 cells, HepG2 cells, and NCM460 cells, thereby enabling a clearer comparison of the uptake variations across the various experimental groups.

### Uptake inhibition studies

RAW264.7, HepG2, and NCM460 cells were seeded in 12-well plates (1 × 10^5^ cells/well) and cultured for 24 h. Cells were pretreated for 1 h with the following inhibitors: amiloride, indomethacin, Pitstop 2, ES9-17, Dynole 2-24, or CPZ. Subsequently, F-GEVs or T-GEVs were added and incubated with the cells for 12 h. Cellular uptake of GEVs was then assessed by flow cytometry to quantify internalization efficiency under each condition. To investigate the contribution of surface proteins, F-GEVs and T-GEVs were subjected to digestion with 0.25% trypsin at a protein-to-enzyme ratio of 2:1 for 1 h at 37 °C. After removing trypsin via ultrafiltration, the resulting particles were designated as DF-GEVs and DT-GEVs, respectively. The fold change in uptake was calculated directly based on the mean fluorescence intensity (MFI) of the reference control using the following formula:

$$\text{Fold change}=\frac{{\mathrm{MFI}}_{1}-{\mathrm{MFI}}_{0}}{{\mathrm{MFI}}_{2}-{\mathrm{MFI}}_{0}}$$ (1)

where MFI_1_, MFI_2_, and MFI_0_ represent the MFI values of the T-GEV, F-GEV, and control groups, respectively.

### In vivo distribution of F-GEVs and T-GEVs

Six-week-old male C57BL/6 mice (body weight 20 ± 2 g) were obtained from Hefei Qingyuan Biotechnology Co. Ltd. After a week of cohabitation, the mice were divided into 2 groups, with 3 replicates per time point. Each group received an oral administration of EVs at a concentration of 5 × 10^9^ particles. The distribution of EVs within the body was subsequently monitored using an in vivo imaging system (IVIS; PerkinElmer, Waltham, USA) at 1, 4, 6, 10, 18, and 24 h post-administration. At each time point, mice from the corresponding group were euthanized, and major organs were excised for ex vivo imaging with IVIS. All animal experiments were approved by the Institutional Animal Care and Use Committee of Hefei University of Technology (protocol no. HFUT20240924001).

### MiRNA detection

The expression levels of several specific miRNAs (cca-miR156b, gma-miR-6300, zma-miR164h-5p, osa-miR-164c, gma-miR396a-3p, aly-miR396a-5p, osa-miR164d, aly-miR159a-3p, and vvi-miR396a) were quantified by RT-qPCR. miRNA sequence information was shown in Table S2. The samples were reverse transcribed using the miRNA first strand cDNA synthesis kit, and 10 pM of synthetic cel-miR-39-3p was added to each sample as a quantitative control. Relative expression was calculated using the 2^−ΔΔCt^ method. Primer sequences were shown in Table S3, and the downstream primer sequence was CGCCCGCCCGCTCCCAAGAT.

### RNA-seq

RNA-seq was performed by OE Biotech Co. Ltd. Libraries were sequenced on an Illumina NovaSeq 6000 platform (150-base pair paired-end). Raw reads were filtered with fastp and aligned to the reference genome using HISAT2, and expression levels were quantified with HTSeq-count. Differential expression analysis was conducted with DESeq2 (*Q* < 0.05 and fold change > 2 or < 0.5 as thresholds for significance). GO and KEGG enrichment analyses were performed to characterize differentially expressed genes (DEGs).

### Preparation of F-GEVs/siRNA^TNF-α^ and T-GEVs/siRNA^TNF-α^

GEVs were loaded with siRNA via electroporation using the Gene Pulser Xcell system (Bio-Rad). Briefly, exosome–siRNA mixtures were transferred into 0.4-cm gap cuvettes and electroporated at varying voltages (280, 300, 320, and 340 V) with a capacitance of 125 μF, resulting in pulse durations of 5 to 10 ms. Following the pulse, samples were incubated on ice for a minimum of 120 min to facilitate membrane recovery. Unincorporated siRNA was subsequently removed by ultrafiltration using 100-kDa Amicon Ultra-0.5 centrifugal filter units at 14,000*g* for 15 min.

### Stability assessment of F-GEVs/siRNA^TNF-α^ and T-GEVs/siRNA^TNF-α^

To evaluate the encapsulation efficiency and integrity of siRNA loaded into F-GEVs and T-GEVs, denaturing PAGE was performed. Samples were prepared under 2 conditions: intact GEVs/siRNA^TNF-α^ and those treated with Triton X-100 to disrupt vesicular membranes. The samples were resolved on a 12% urea–polyacrylamide gel at 110 V for 30 min. Post-electrophoresis, the gel was imaged using a Tanon MINI Space 3000 system.

### Evaluation of therapeutic effects

BALB/c mice were randomly allocated into 6 groups: (a) healthy control (PBS); (b) DSS model (4.0% DSS; PBS); (c) DSS + F-GEVs (5 × 10^9^ particles); (d) DSS + T-GEVs (5 × 10^9^ particles); (e) DSS + F-GEVs/siRNA^TNF-α^ (30 nmol/kg siRNA); and (f) DSS + T-GEVs/siRNA^TNF-α^ (30 nmol/kg siRNA). To evaluate the severity of colitis, the DAI was calculated as the summation of scores for body weight loss, stool consistency, and rectal bleeding (scale, 0 to 4). Mice were euthanized on day 14, at which point colons and major organs were harvested for histopathological analysis. Colon, kidney, and liver tissues were fixed in formalin, embedded in paraffin, and subsequently stained with H&E. All animal experiments were approved by the Institutional Animal Care and Use Committee of Hefei University of Technology (protocol no. HFUT20240924001).

### Quantitative real-time polymerase chain reaction

Total RNA was extracted using TRIzol reagent following the manufacturer’s protocol, reverse transcribed, and quantified using standard RT-qPCR procedures. Primer sequences are provided in Table S4.

### Statistics

The experimental results were performed using GraphPad Prism, with values expressed as mean ± standard deviation (SD). Data were subjected to one-way analysis of variance (ANOVA) analysis, and statistical significance was determined at *P < 0.05, **P < 0.01, ***P < 0.001, ****P < 0.0001, with ns indicating no significance.

## Data Availability

The datasets underpinning the results of this research can be obtained from the corresponding authors upon a justified request.
