# Supplementary material for: Thermally Induced Reassembly of Ginger Extracellular Vesicles for Oral Therapy of Intestinal Inflammation
Source: Research (Wash D C). 2026 Aug 3;9:1377. doi: 10.34133/research.1377 (PMC13429916; doi:10.34133/research.1377)
Supplement: Supplementary 1 — Figs. S1 to S19 Tables S1 to S4 [file research.1377.f1.docx]

**Supplementary Materials**

**Thermally-Induced Reassembly of Ginger Extracellular Vesicles for Oral Therapy of Intestinal Inflammation**

Linhai Hou ^1^, Jie Cao ^1^, Shengjie Gao^1^, Xiaofan Wang ^1^, Zhongxian Zhang ^1^, Meiqi Li^.1^, Yu Mao ^1^, Changhong Liu ^1^, Ling Yan ^1,3 *^, Haiping Hao ^2 *^, Lei Zheng ^1,3 *^

^1^School of Food and Biological Engineering, Hefei University of Technology, Hefei 230009, China.

^2^ State Key Laboratory of Natural Medicines, Key Laboratory of Drug Metabolism, China Pharmaceutical University, Nanjing, China.

^3^ Engineering Research Center of Bio-Process, Ministry of Education, School of Food and Biological Engineering, Hefei University of Technology, Hefei 230009, China.

* Corresponding authors.

School of Food and Biological Engineering, Hefei University of Technology, Hefei 230009, China. State Key Laboratory of Natural Medicines, Key Laboratory of Drug Metabolism, China Pharmaceutical University, China.

Email: 2019800096@hfut.edu.cn (Ling Yan), [haipinghao@cpu.edu.cn](mailto:haipinghao@cpu.edu.cn) (Haiping Hao), [lzheng@hfut.edu.cn](mailto:lzheng@hfut.edu.cn) (Lei Zheng).


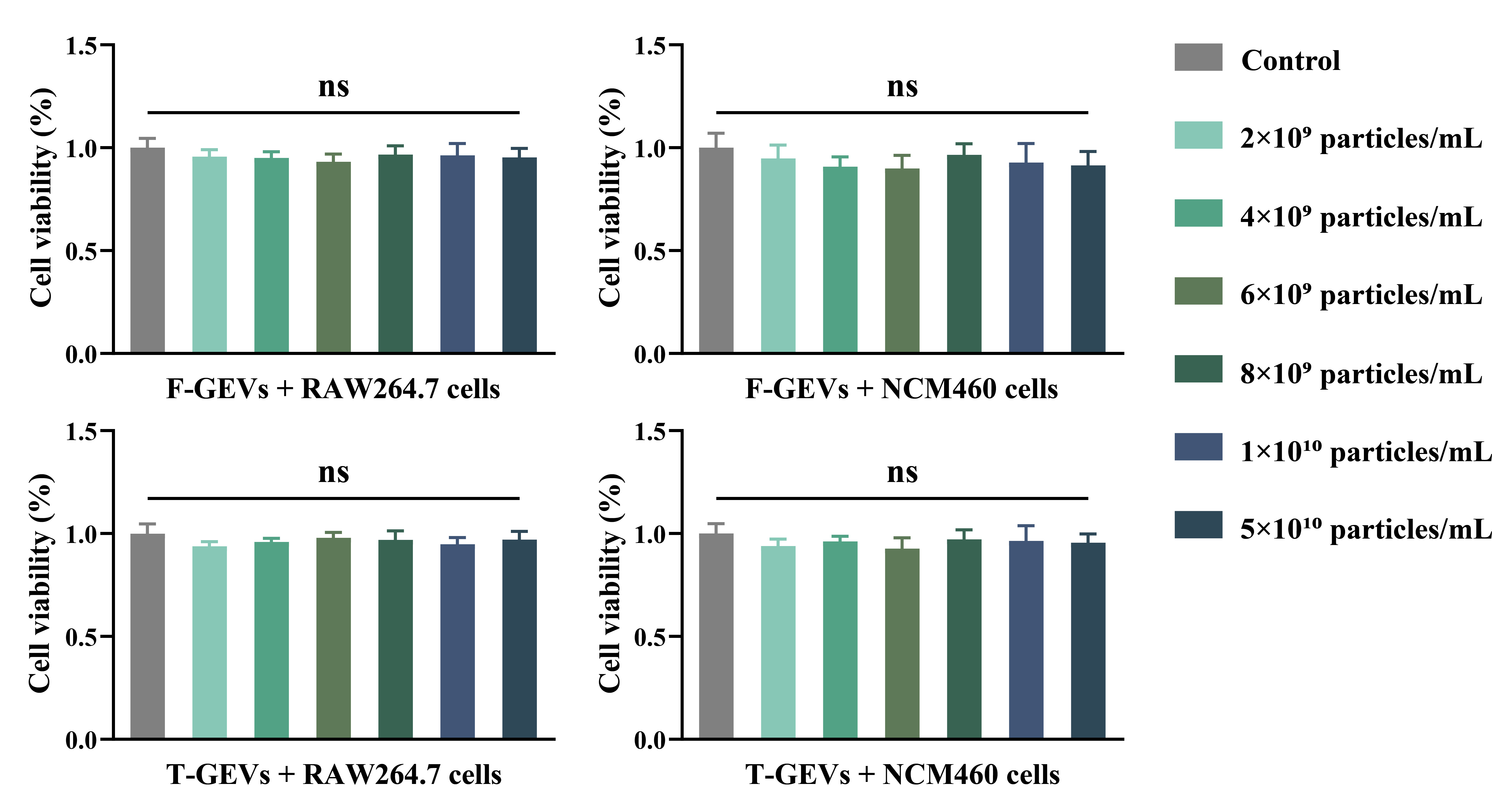


**Fig. S1. Cell viability of RAW264.7, NCM460, and HepG2 cells after 24 h treatment with F-GEVs and T-GEVs.** All data are presented as means ± SD, n = 6. P values were calculated using two-sided one-way ANOVA post-Dunnett’s test; ns, non-significant.


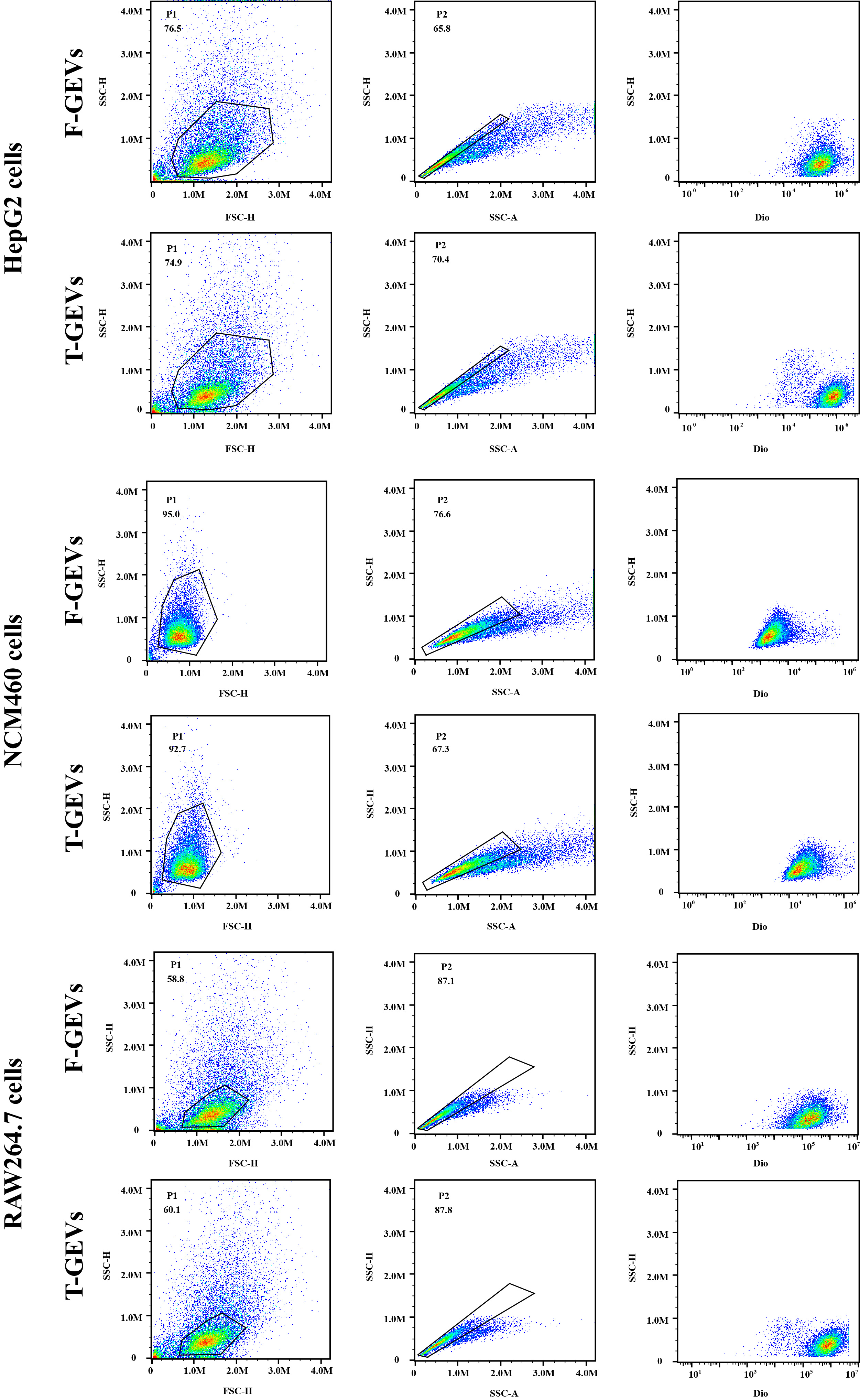


**Fig. S2. Flow cytometry gating strategy for analysis of F-GEVs and T-GEVs uptake in HepG2, NCM460, and RAW264.7 cells.**


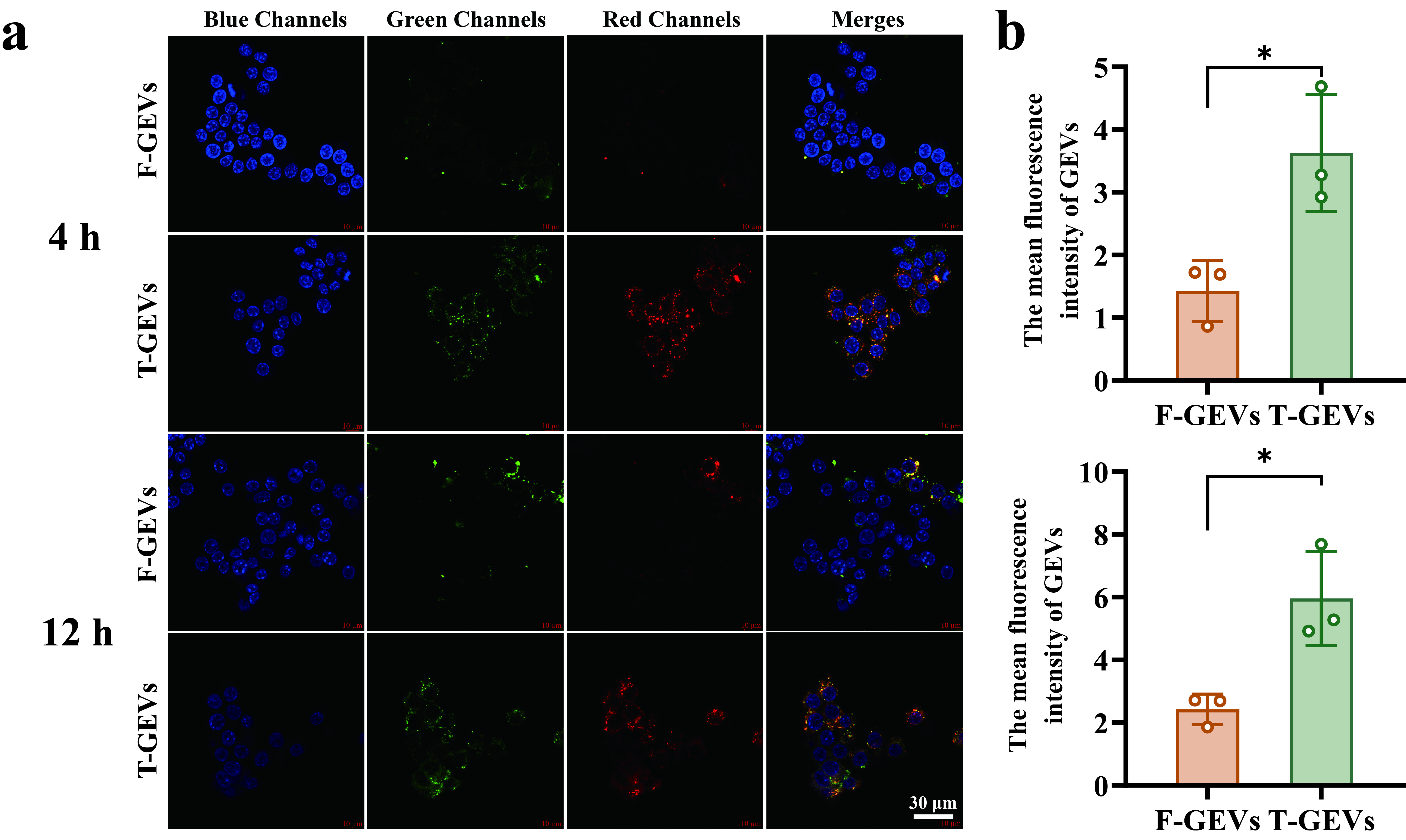


**Fig. S3. Uptake of T-GEVs or F-GEVs in RAW264.7 cells detected by confocal microscopy (a-b).** **Blue: nucleus; Green: cell membrane; Red: F-GEVs or T-GEVs.** All data are presented as means ± SD, n = 3. P values were calculated using two-sided one-way ANOVA post-Dunnett’s test; **P* < 0.05.


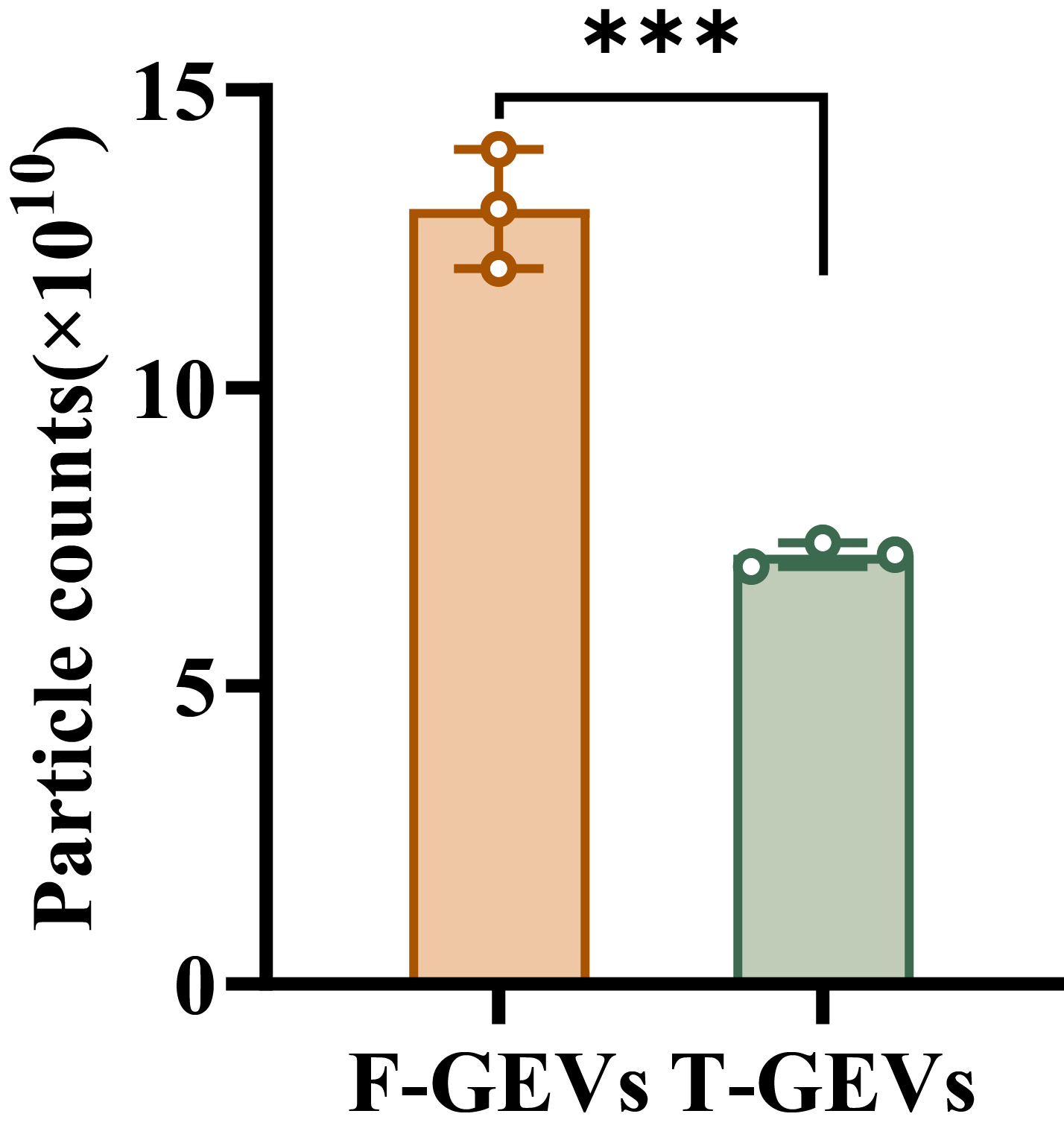


**Fig. S4. The particle counts of F-GEVs and T-GEVs determined by NTA.** All data are presented as means ± SD, n = 3. P values were calculated using two-sided one-way ANOVA post-Dunnett’s test; ****P* < 0.001.


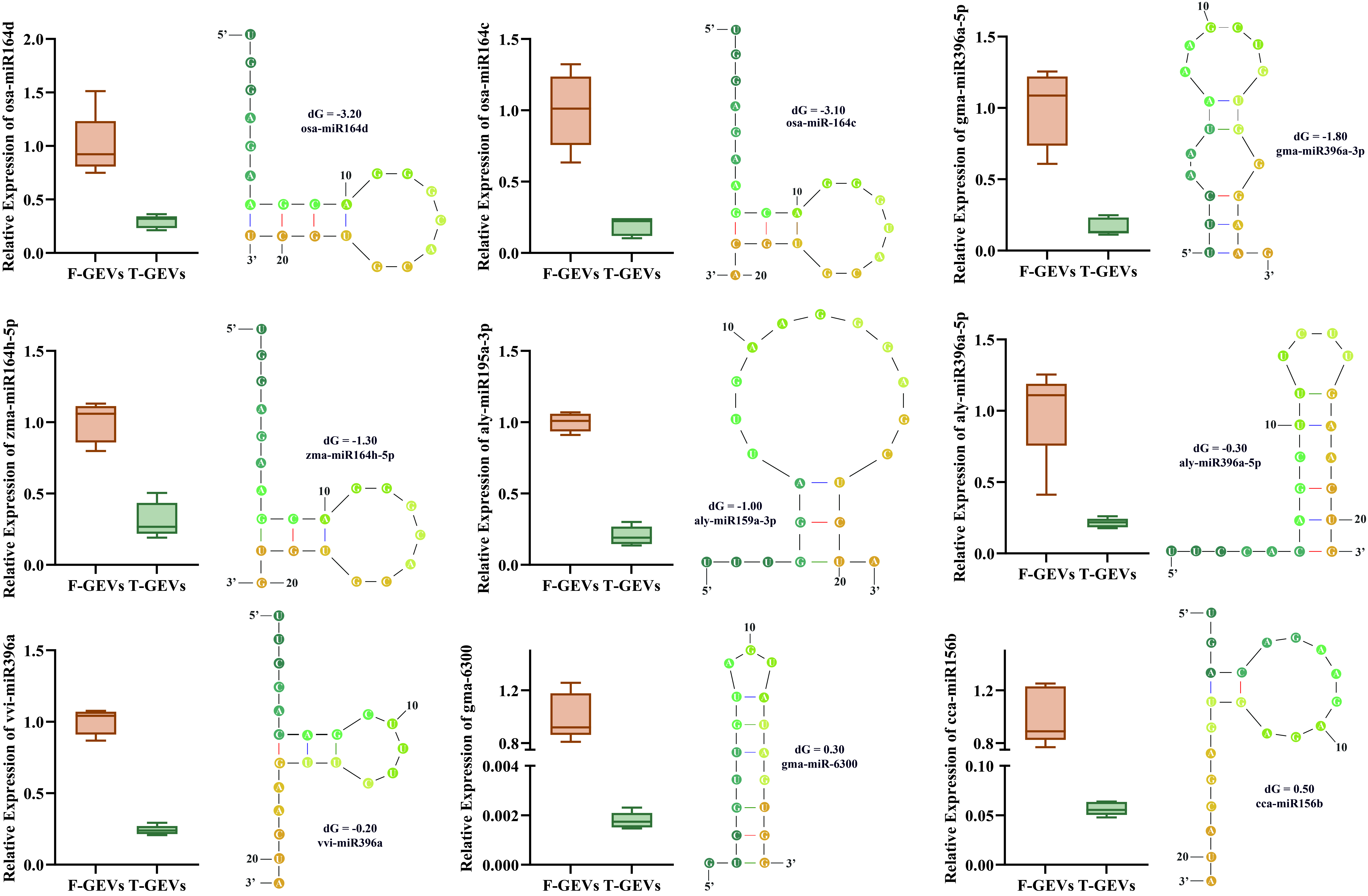


**Fig. S5. Relative levels of selected miRNAs found in T-GEVs after boiling.** Relative expression of selected plant miRNAs in F-GEVs and T-GEVs by RT–qPCR (left). Predicted secondary structures with minimum free energy (ΔG) were shown (right). All miRNAs are more abundant in F-GEVs than in T-GEVs.


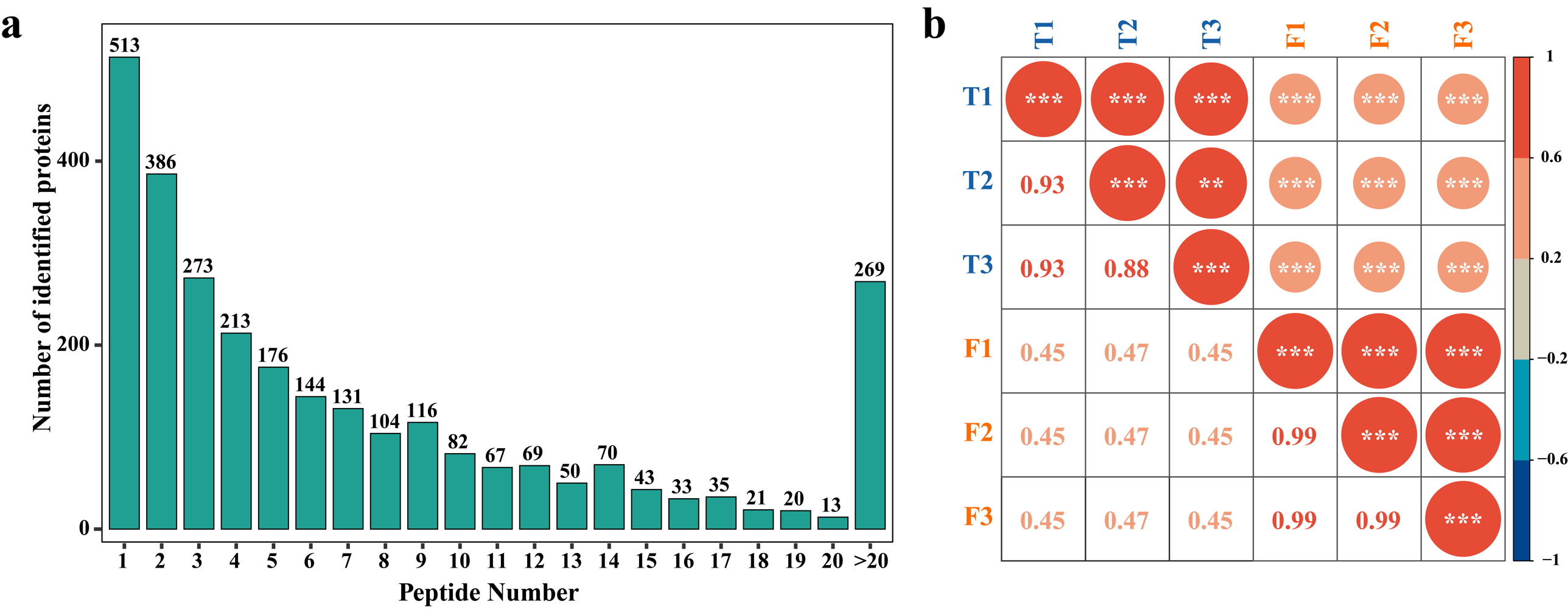


**Fig. S6.** **Mass error analysis and correlation analysis of the DAI method based on the LC-MS/MS proteomics workflow**. (a), Characterizing the distribution of peptide lengths. (b), Correlation analysis on the different samples for F-GEVs (F1, F2, F3) and T-GEVs (T1, T2, T3).


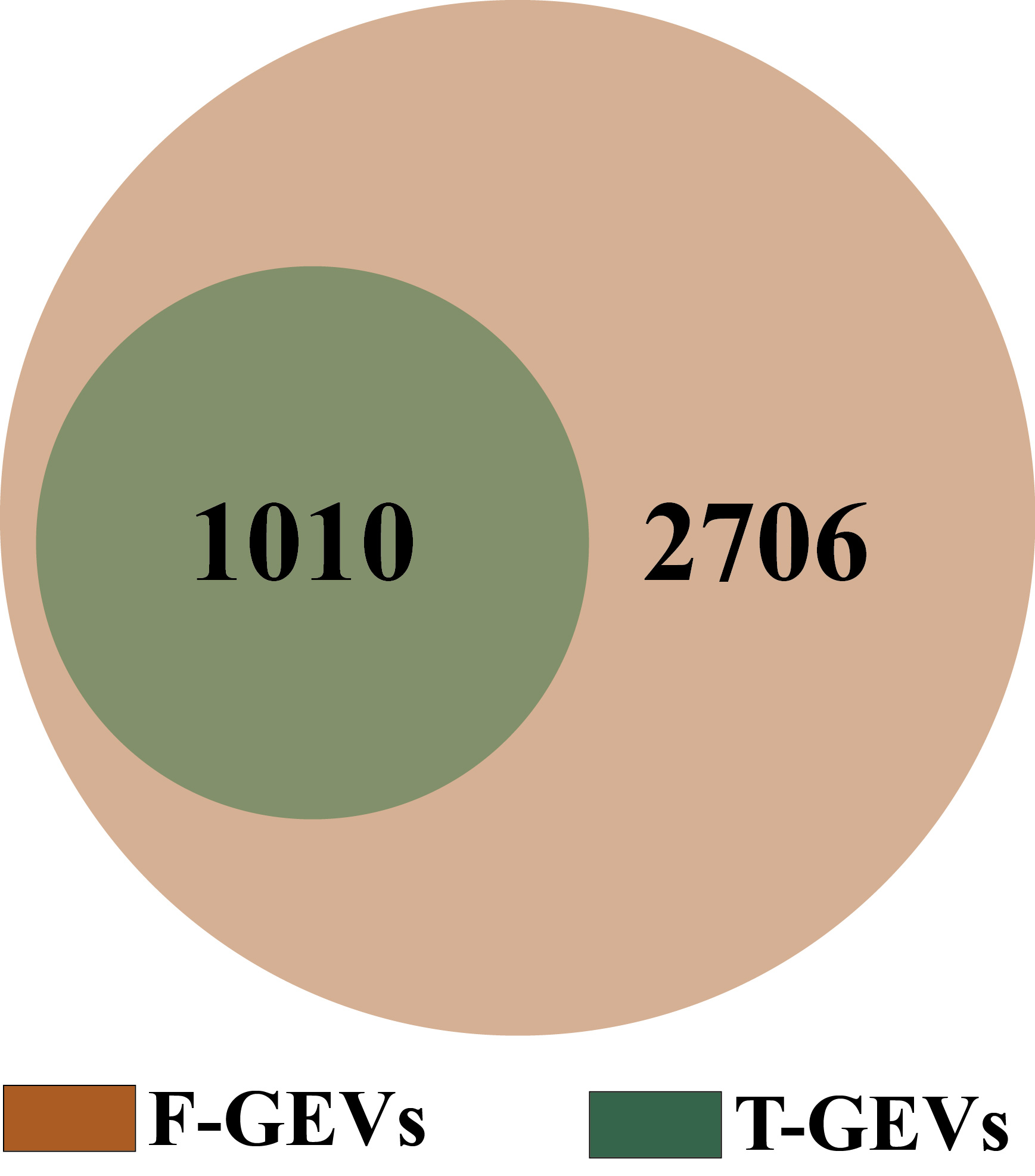


**Fig. S7.** **Venn diagram comparing significantly altered proteins as identified.**


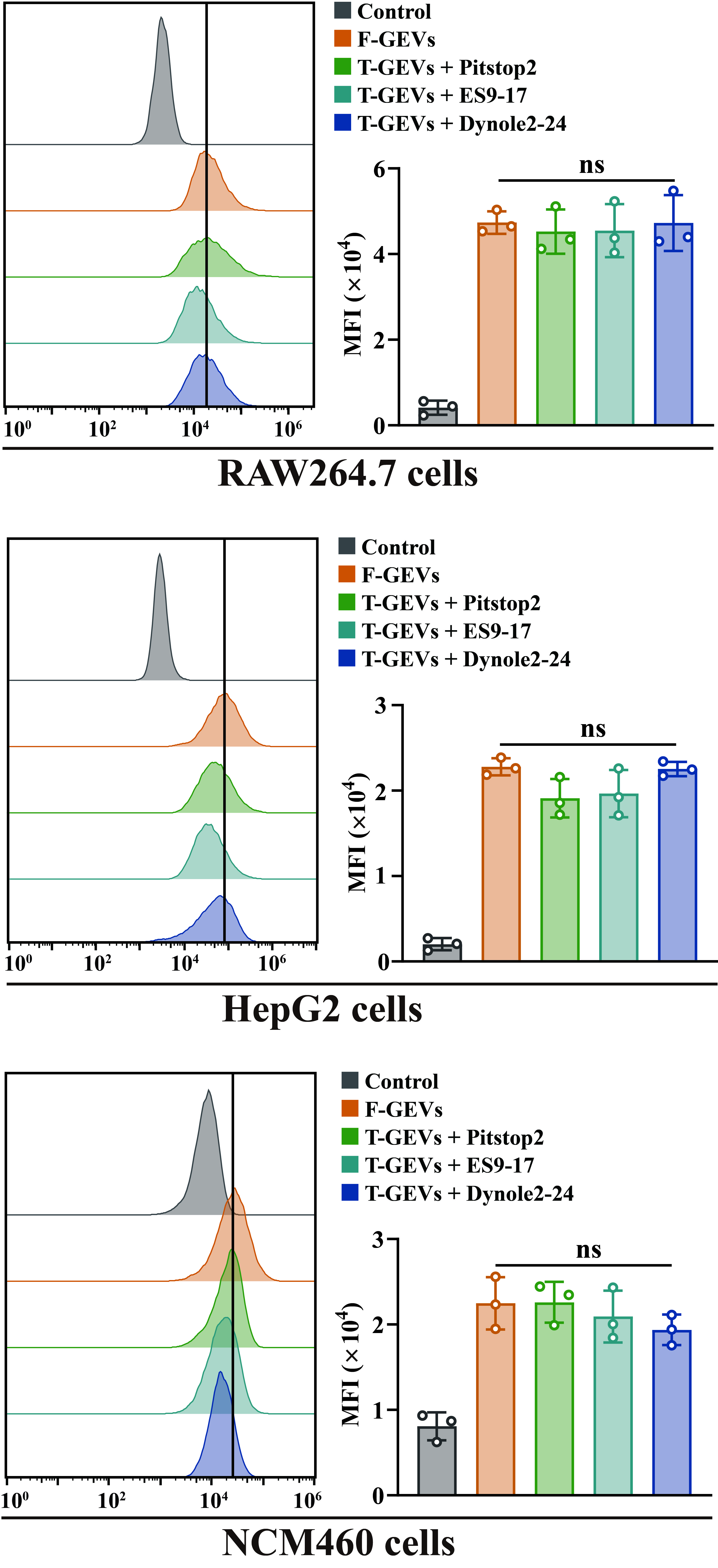


**Fig. S8.** **Cellular uptak****e of T-GEVs under different inhibitors of clathrin-mediated endocytosis.** All data are presented as means ± SD, n = 3. P values were calculated using two-sided one-way ANOVA post-Dunnett’s test; ns, non-significant.


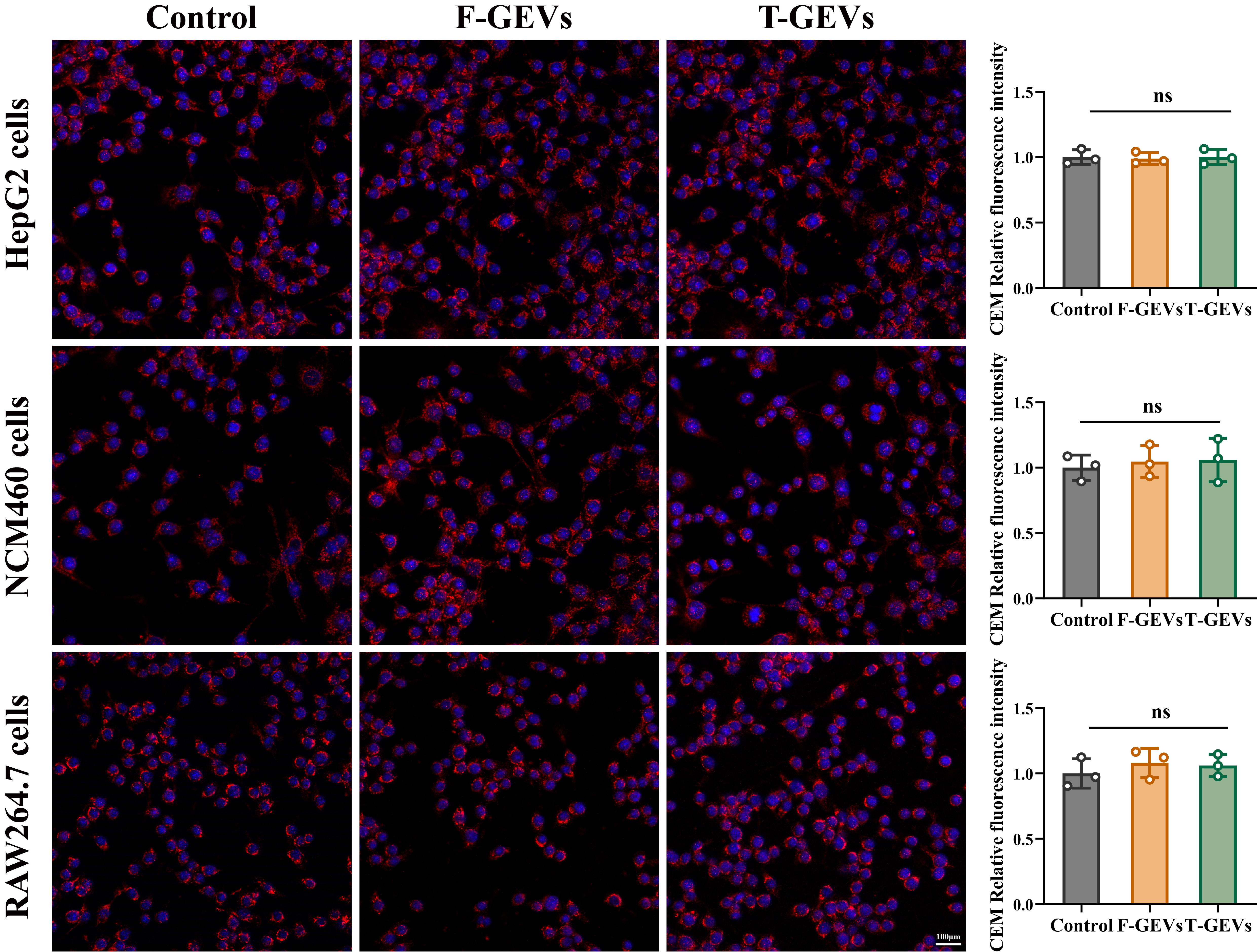


**Fig. S9.** **Immunofluorescence analysis of membrane-associated clathrin expression in HepG2 cells, NCM460 cells, and RAW264.7 cells**. Scale bars, 100 μm. All data are presented as means ± SD, n = 3. P values were calculated using two-sided one-way ANOVA post-Dunnett’s test; ns, non-significant.


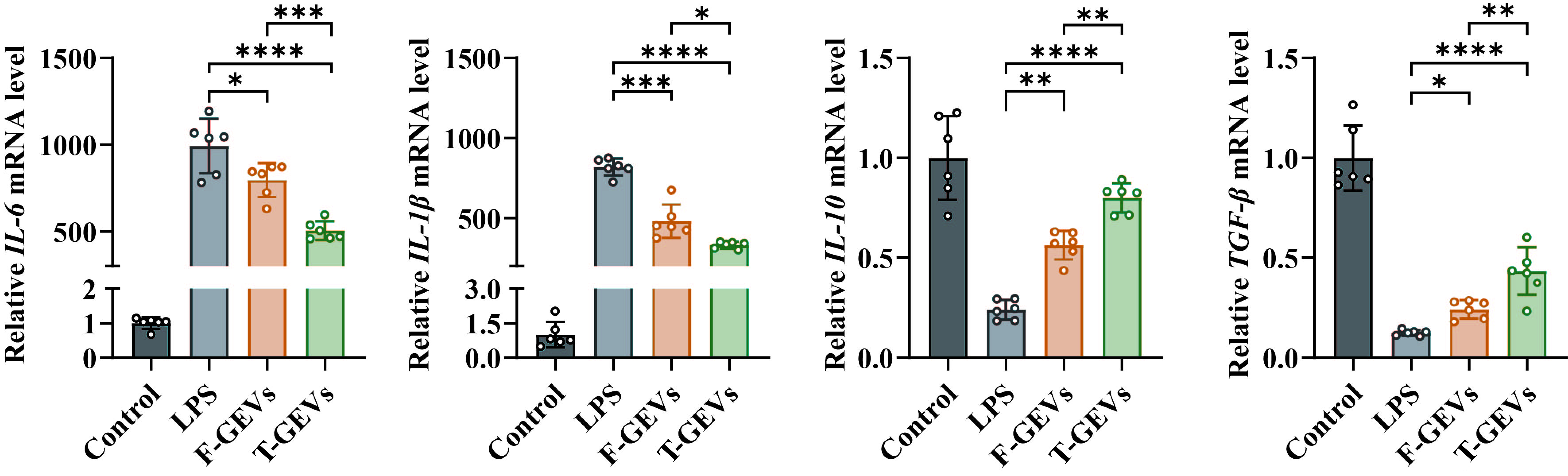


**Fig. S10.** **The mRNA expression levels of *IL-6*, *IL-1β*, *TGF-β*, and *IL-10* were measured by RT-qPCR.**


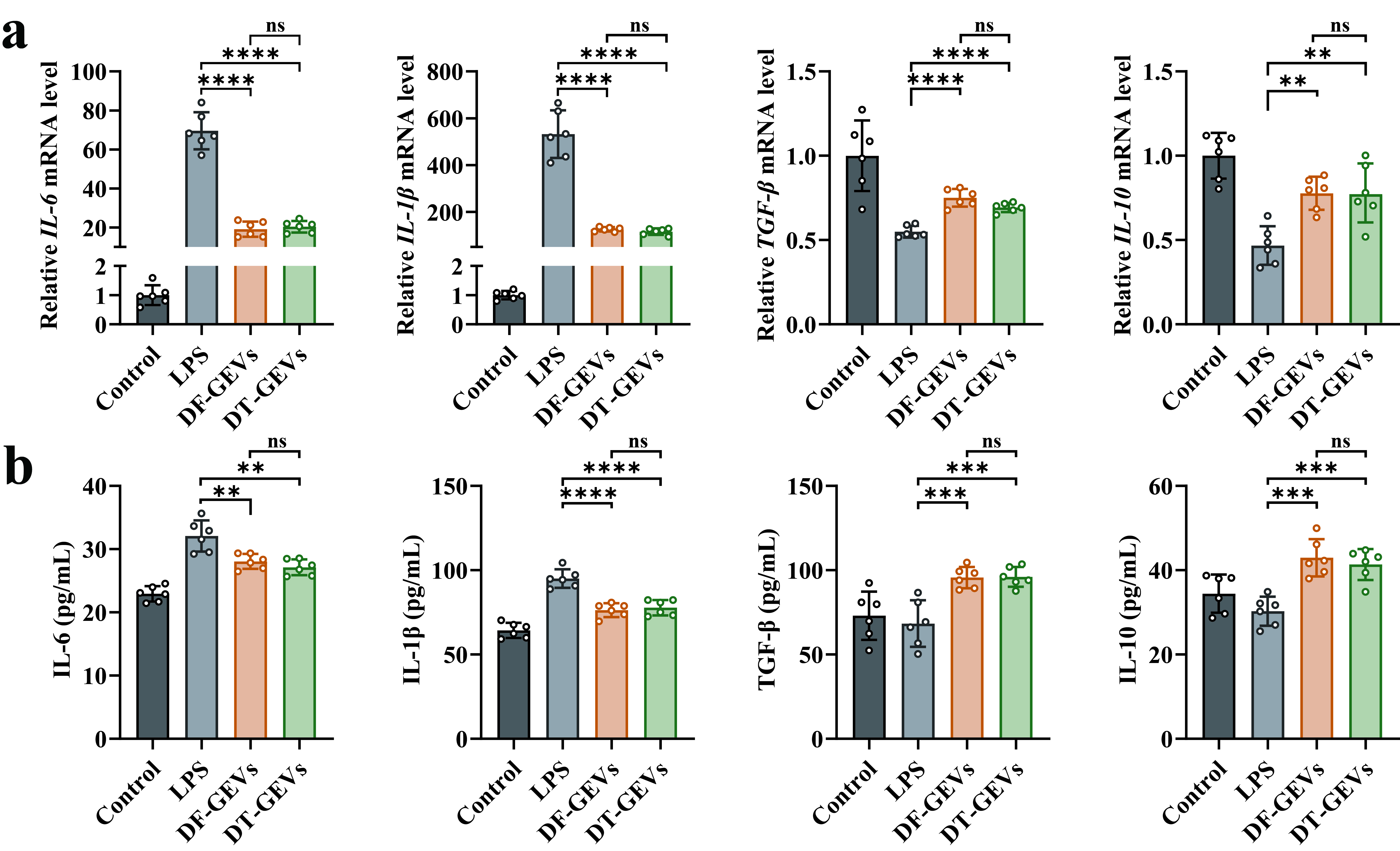


**Fig. S11.** **Anti-inflammatory effect of DF-GEVs and DT-GEVs *in vitro***. (a), The mRNA expression levels of *IL-6*, *IL-1β*, *TGF-β*, and *TNF-α* were measured by RT-qPCR (normalized to GAPDH, n=6 biological replicates). (b), ELISA analysis showing the levels of inflammatory cytokines and TGF-β in macrophages transfected with F-GEVs and T-GEVs. All data are presented as means ± SD, n = 6. P values were calculated using two-sided one-way ANOVA post-Dunnett’s test; ***P* < 0.01, ****P* < 0.001, *****P* < 0.0001, ns, non-significant.


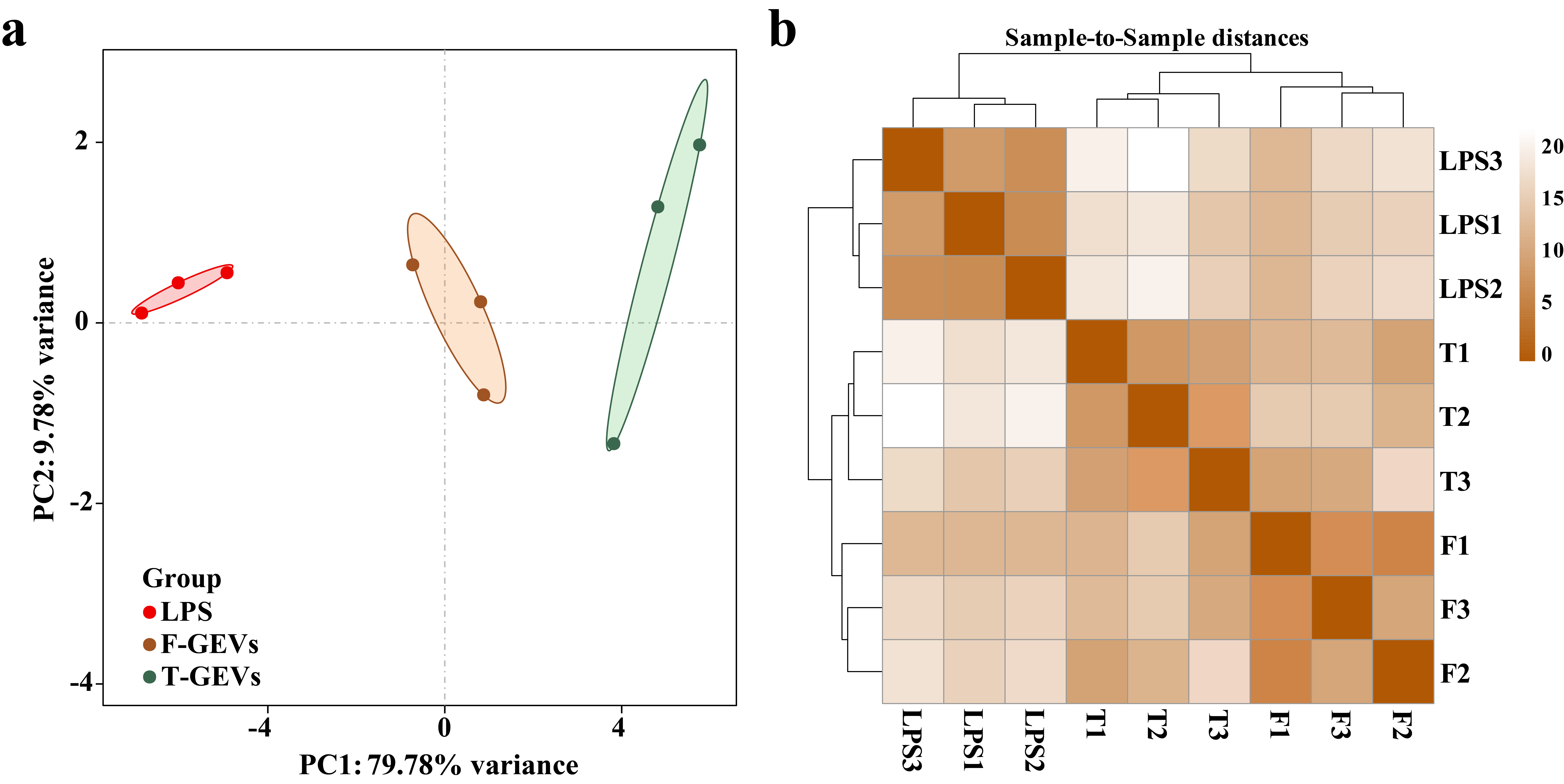


**Fig. S12.** **Evaluation of biological replicates and inter-sample correlations.** (a), Principal component analysis (PCA) of 9 transcriptomes. (b), Hierarchical clustering heatmap illustrating inter-sample relationships.


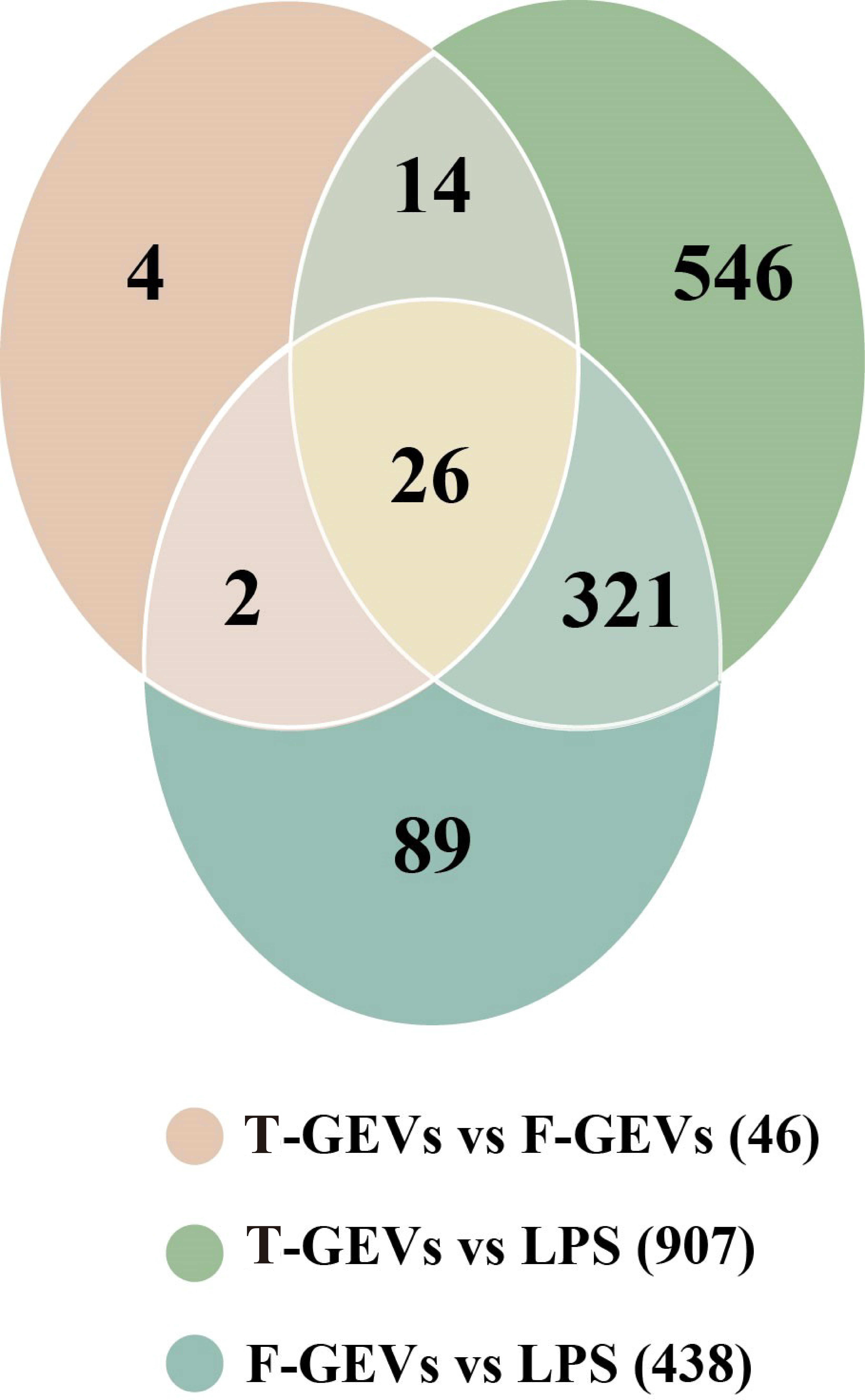


**Fig. S13. Venn diagrams visually illustrated the shared and unique differentially expressed genes across the comparison groups.**


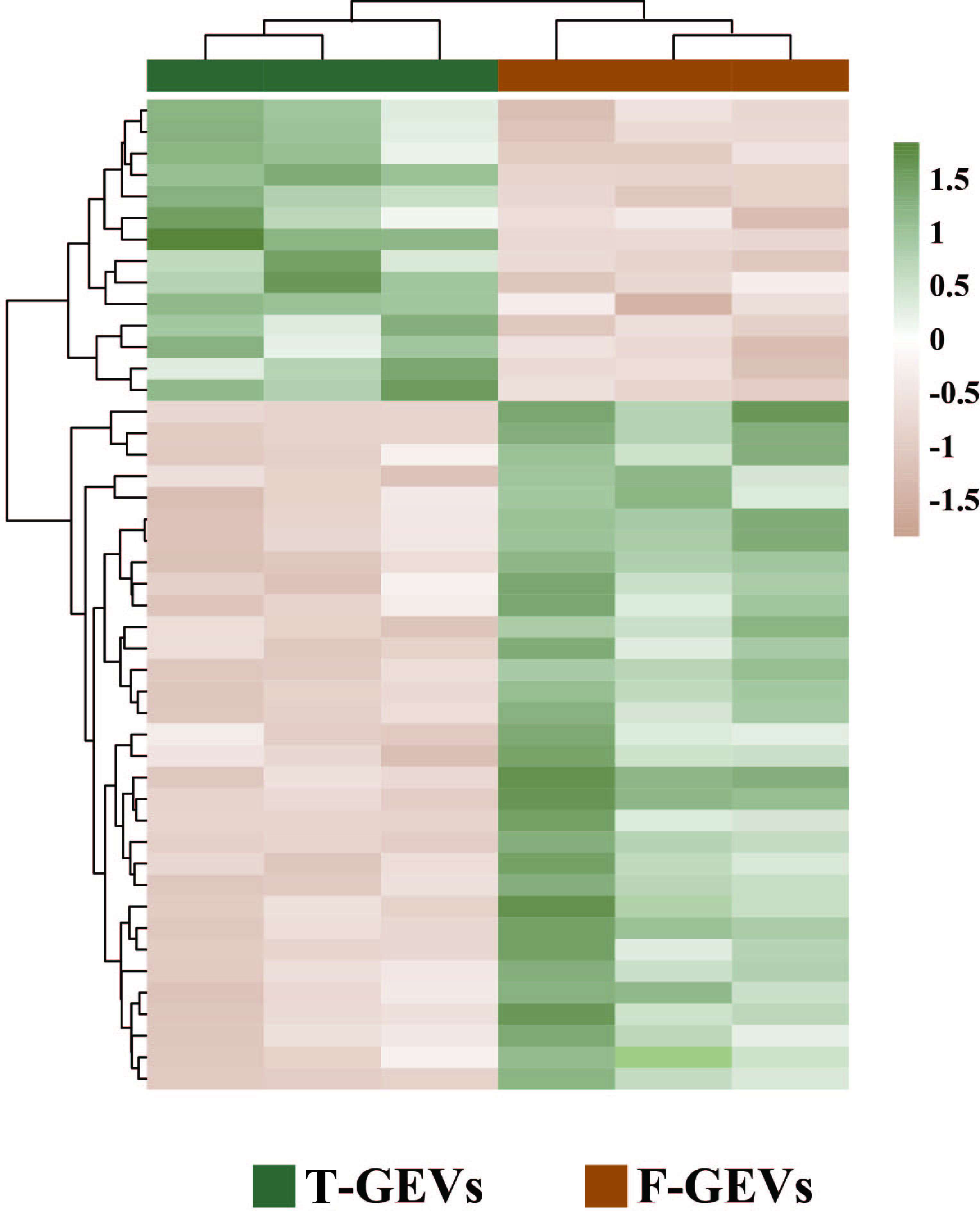


**Fig. S14. Heat map of key gene expression showing differentially expressed genes between F-GEVs and T-GEVs.**





**Fig. S15. Downregulation of inflammatory genes in the T-GEVs treatment group.** (a, c, e) Comparison of differentially expressed genes between the F-GEVs and LPS group (a), the T-GEVs and LPS group (c), and the F-GEVs and T-GEVs group (e). (b, d, f) Top 10 gene GO terms enriched in downregulated genes in (b) the F-GEVs vs LPS group, (d) the T-GEVs vs LPS group, and (f) the F-GEVs vs T-GEVs.



**Fig. S16. GO and KEGG pathway enrichment analyses of up-regulated genes in T-GEVs.** (a), Top 30 significantly enriched GO terms for genes upregulated in T-GEVs, spanning biological processes (blue), cellular components (green), and molecular functions (purple). (b), KEGG pathway classification showing upregulated (orange) and downregulated (green) pathways.


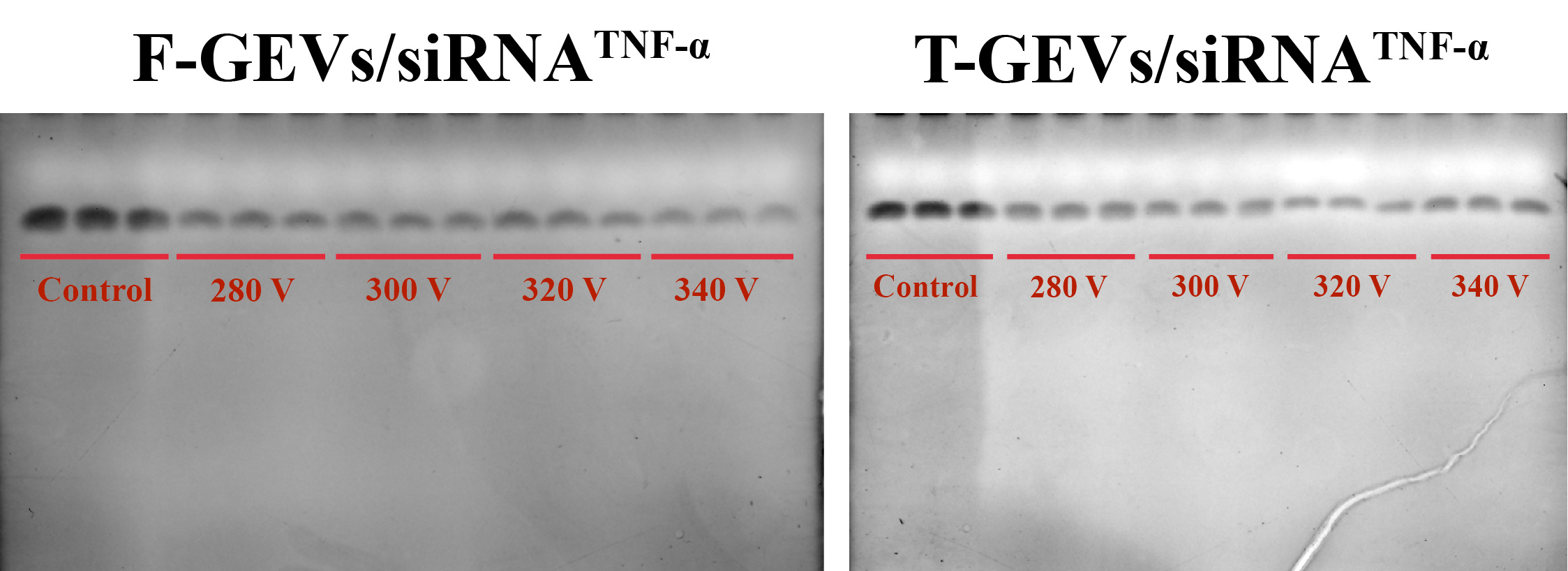


**Fig. S17. RNA gel assay for determining siRNA encapsulation efficiency in F-GEVs/siRNA^TNF-α^ and T-GEVs/siRNA^TNF-α^.**


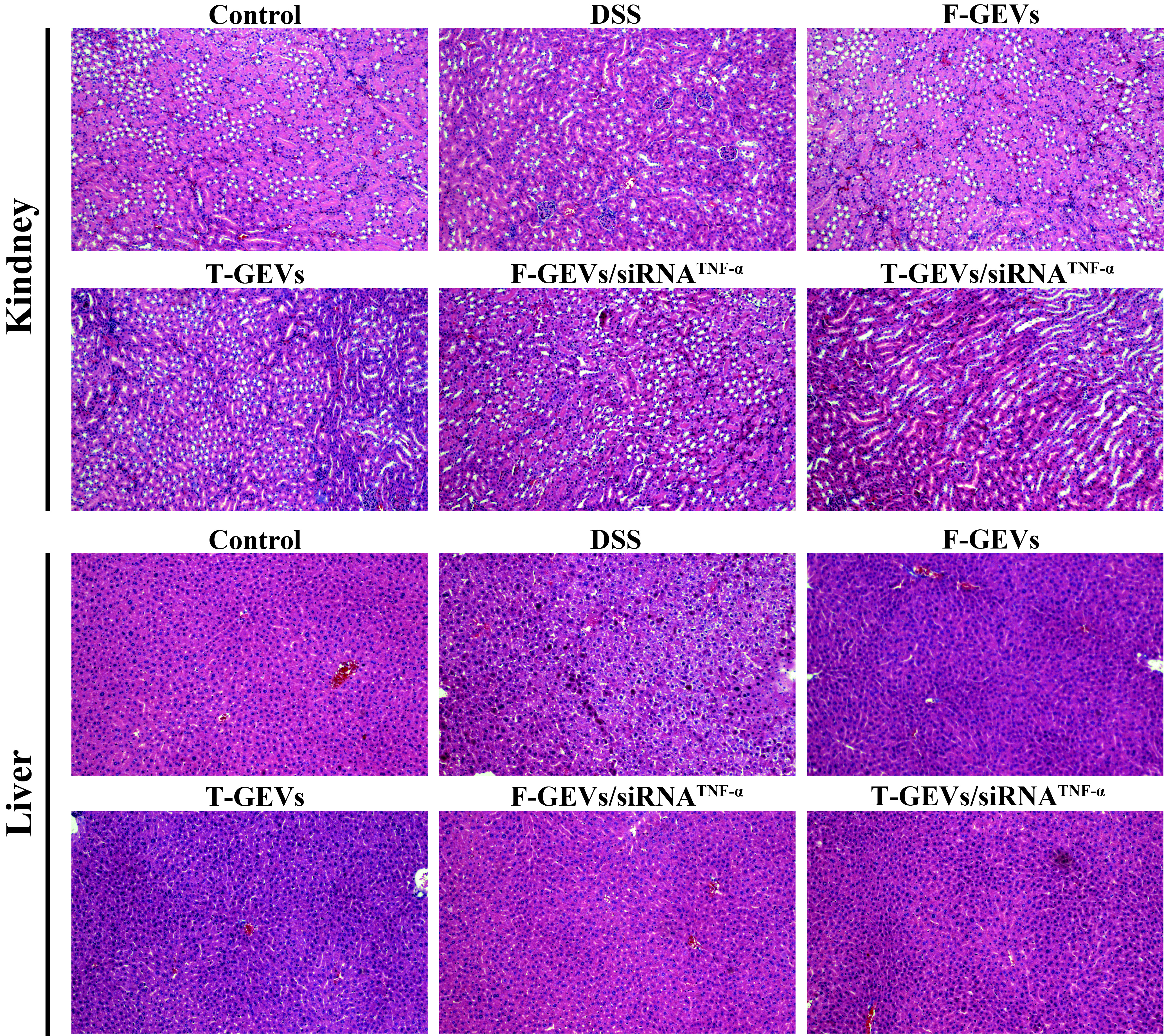


**Fig. S18.** **Representative H&E-stained histological sections of kidney and liver tissues.**


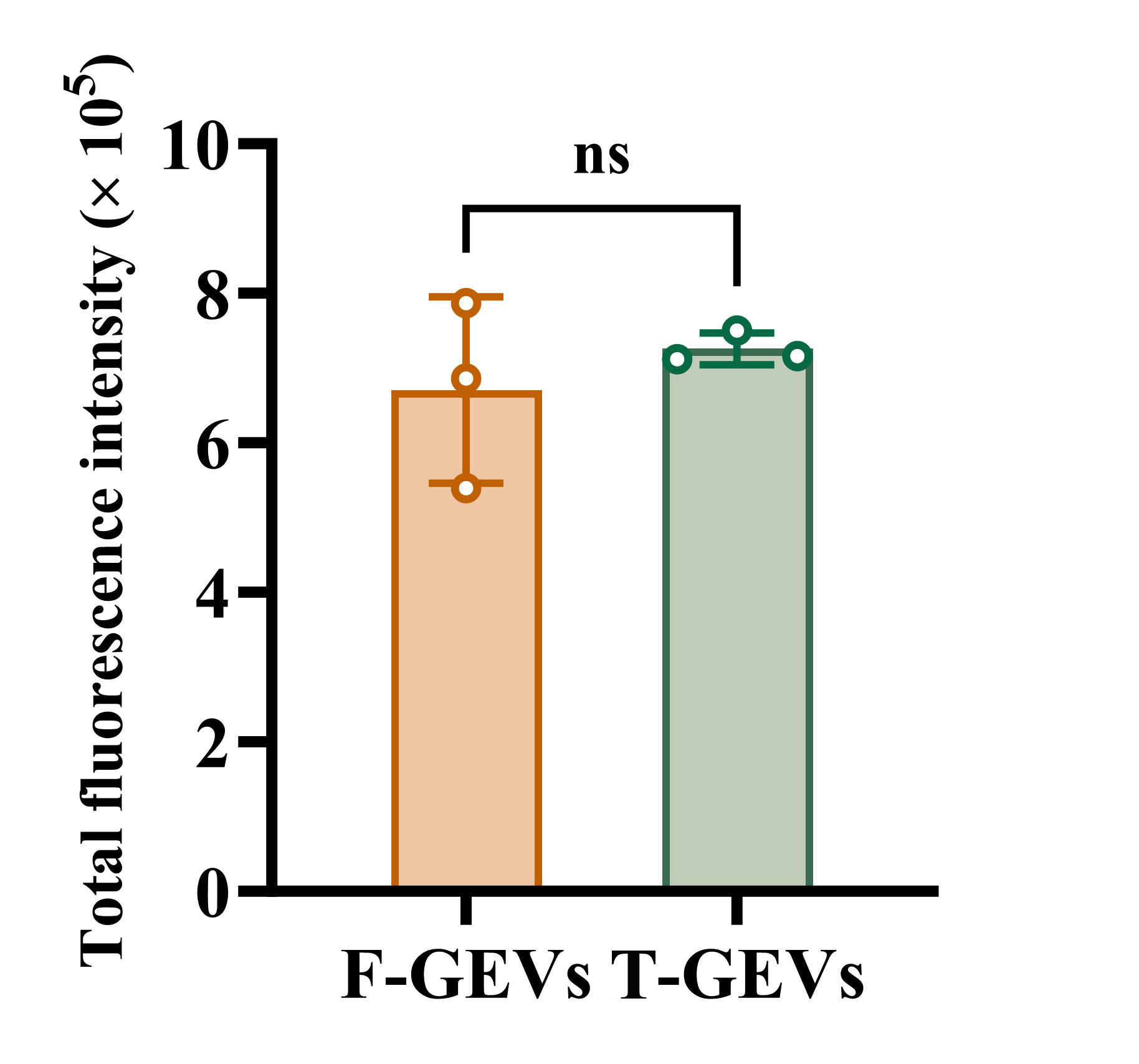


**Fig. S19.** **Quantitative analysis of fluorescence intensity from DIO-labeled F-GEVs and T-GEVs.** All data are presented as means ± SD, n = 3. P values were calculated using two-sided one-way ANOVA post-Dunnett’s test; ns, non-significant.

**Supplementary Table 1.** **Proteomic profile of significantly up-regulated proteins in T-GEVs compared to F-GEVs.**

| Categories | Accession ID | Number of entries |
| --- | --- | --- |
| Iron Storage Proteins | A0A8J5C078_, A0A8J5GJQ7, A0A8J5C649, A0A8J5C3W0 | 4 |
| Enzymes | A0A8J5H1I9, A0A8J5H5T2, A0A8J5I0Y3, A0A8J5FJR2, A0A8J5GL54, A0A8J5ESJ0, A0A8J5H5L4, A0A8J5HFV4, A0A8J5EW52, A0A8J5KZW9, A0A8J5FEZ1, A0A8J5FZX4, A0A8J5GQ98, A0A8J5GE67, A0A8J5FCX5, A0A8J5F4N3, A0A8J5HY50, A0A8J5F1R5, A0A8J5G393, A0A8J5L7C2, A0A8J5HGC2, A0A8J5F8F4, A0A8J5KEC2, A0A8J5FAP7, A0A8J5G2E0, A0A8J5G723, A0A8J5FWJ6, A0A8J5LKQ6 | 28 |
| Cytoskeleton and structural proteins | A0A8J5HSA3, A0A8J5C5Z1 | 2 |
| Metabolism-related protein | 0A8J5M889, A0A8J5H7F0, A0A8J5HYQ2, A0A8J5G842, A0A8J5G706, A0A8J5HY50, A0A8J5F1R5, A0A8J5G393, A0A8J5L7C2, A0A8J5HGC2, A0A8J5F8F4, A0A8J5KEC2, A0A8J5FAP7, A0A8J5G2E0, A0A8J5G723 | 15 |
| Transporter and membrane proteins | A0A8J5C7J5, A0A8J5BUW5, A0A8J5HMA7, A0A8J5HVS4, A0A8J5CHX9, A0A8J5G7S6, A0A8J5KMM5, A0A8J5I2Z3, A0A8J5EX28, A0A8J5GXD2, A0A8J5HN05, A0A8J5FT37 | 12 |
| Proteases and Inhibitors | A0A8J5LHG8, A0A8J5HXU1, Q5ILG7, A0A8J5GGN5, A0A8J5GYR8, A0A8J5H2Y1, A0A8J5I9T8, A0A8J5EAI2, A0A8J5LFA9 | 9 |
| Signal transduction and regulatory proteins | A0A8J5GZ21, A0A8J5IBG6, A0A8J5GBB3, A0A8J5FLB3, A0A8J5G2E5, A0A8J5G0R2, A0A8J5KRX2, A0A8J5LQN1, A0A8J5GB40, A0A8J5INA7, A0A8J5KL83 | 11 |
| Immunity and defense-related proteins | A0A8J5FY09, A0A8J5KSJ4, A0A8J5FB99, A0A8J5FVG1, A0A8J5L4N4, A0A8J5F2D5, A0A8J5EXW3, A0A8J5GW31, A0A8J5GUY2, A0A8J5KDK3, A0A8J5F1A6, A0A8J5EXT5, A0A8J5EZ66, A0A8J5F5Y1, A0A8J5F663, A0A8J5I0E8, A0A8J5HI52, A0A8J5FVS5, A0A8J5C4N1, A0A8J5G2N9 | 20 |
| Redox and antioxidant proteins | A0A8J5I4A7, A0A8J5KMR2, A0A8J5KU86, A0A8J5GIW4, A0A8J5HCU4, A0A8J5G393, A0A8J5LKQ6, A0A8J5FWJ6 | 8 |
| Molecular chaperones and folding-associated proteins | A0A8J5G7W0, A0A8J5EW23, A0A8J5GKM2, A0A8J5HN05, A0A8J5GFF6, A0A8J5G9K1, A0A8J5C933 | 7 |
| Nucleic acid metabolism-related proteins | A0A8J5BX78, A0A8J5EZD5, A0A8J5FZU0, A0A8J5FS06, A0A8J5C8S5, A0A8J5HQF3, A0A8J5GF55, A0A8J5M7M9, A0A8J5HPS3, A0A8J5ER13 | 10 |
| Multifunctional complex subunit | A0A8J5CEV1, A0A8J5I688, A0A8J5G9K1, A0A8J5FT37, A0A8J5G7S6, A0A8J5CHX9 | 6 |
| Other functionally defined proteins | A0A8J5BUC7 (atpA), A0A8J5BA52 (atpB), A0A8J5GXR6, A0A8J5FBV8, A0A8J5HI46 (CYN), A0A8J5FY97, A0A8J5GZ76, A0A8J5GYZ1, A0A8J5GSD3, A0A8J5BCW2, A0A8J5GG59, A0A8J5BYP6, A0A8J5C5Z8 | 13 |
| Uncharacterized Protein | A0A8J5F812, A0A8J5HCL0, A0A8J5FQM2, A0A8J5LDP7, A0A8J5LNZ5, A0A8J5GIA4, A0A8J5FKI9, A0A8J5F8J5, A0A8J5C6U5, A0A8J5I445, A0A8J5F8H1, A0A8J5LCZ3, A0A8J5L8P8, A0A8J5FZU8, A0A8J5GFF6, A0A8J5L717, A0A8J5HTA2, A0A8J5F3S8, A0A8J5FJR2 | 19 |

**Supplementary Table 2. Sequence of miRNA mimics.**

| miRNA | sequence（5’-3’） |
| --- | --- |
| cca-miR156b | UGACAGAAGAGAGUGAGCAUA |
| gma-miR-6300 | GUCGUUGUAGUAUAGUGG |
| osa-miR-164c | UGGAGAAGCAGGGUACGUGCA |
| zma-miR164h-5p | UGGAGAAGCAGGGCACGUGUG |
| gma-miR396a-3p | UUCAAUAAAGCUGUGGGAAG |
| aly-miR396a-5p | UUccacagcUUUcUUgaacUg |
| osa-miR164d | UGGAGAAGCAGGGCACGUGCU |
| aly-miR159a-3p | UUUggaUUgaagggagcUcUa |
| vvi-miR396a | UUCCACAGCUUUCUUGAACUA |

**Supplementary Table 3. Primer sequence of the GEVs-derived miRNA.**

| miRNA | sequence（5’-3’） |
| --- | --- |
| cca-miR156b | ccgtgacagaagagagtgagcata |
| gma-miR-6300 | cgccgtcgttgtagtatagtgg |
| osa-miR-164c | attggagaagcagggtacgtg |
| zma-miR164h-5p | aatatggagaagcagggcacgtgt |
| gma-miR396a-3p | ccgcttcaataaagctgtgggaag |
| aly-miR396a-5p | cggttccacagctttcttgaactg |
| osa-miR164d | Aatattggagaagcagggcacgt |
| aly-miR159a-3p | ccgtttggattgaagggagctcta |
| vvi-miR396a | CGCTTCCACAGCTTTCTTGAACTA |
| cel-miR-39-3p | TCACCGGGTGTAAATCAGCTTG |

**Supplementary Table 4. Primer sequences for RT-qPCR.**

| Gene | primer sequence（5’-3’） |
| --- | --- |
| IL-1β | Forward-GCAGAGCACAAGCCTGTCTTCC  Reverse-ACCTGTCTTGGCCGAGGACTAAG |
| IL-6 | Forward-AATTTCCTCTGGTCTTCTGGAGT  Reverse-GTGACTCCAGCTTATCTCTTGGT |
| IL-10 | Forward-TTCTTTCAAACAAAGGACCAGC  Reverse-GCAACCCAAGTAACCCTTAAAG |
| TGF-β | Forward-GCATTGGCAAAGGTCGGTTT  Reverse-TGCCTCTCGGAACCATGAAC |
| TNF-α | Forward-GCATGATCCGAGATGTGGAACTGG  Reverse-CGCCACGAGCAGGAATGAGAAG |
| NLRP3 | Forward-AAACCCACCAGTGTGCAAGA  Reverse-CAAAGGCCCCTTGTAGCTCA |
| Gbp2 | Forward-GCAAACCCTGGTTCTGCTTG  Reverse-CACATAGTGCAGCTGGTCCA |
| Gbp7 | Forward-AAGGGCATCTGGATGTGGTG  Reverse-ATCTCCTAAGCCCTCCGTGT |
| IL-18 | Forward-ACGGCAAGACCAAGACTCTG  Reverse-GTCACTGCGTTCTCCAGACA |
| GAPDH | Forward-AGGTCGGTGTGAACGGATTTG  Reverse- AGAAGGGTCACTCAGGATAA |
